# Canonical Structure and Orthogonality of Forces and Currents in Irreversible Markov Chains

**DOI:** 10.1007/s10955-018-1986-0

**Published:** 2018-02-15

**Authors:** Marcus Kaiser, Robert L. Jack, Johannes Zimmer

**Affiliations:** 1 Department of Mathematical Sciences, University of Bath, Bath, BA2 7AY UK; 2 Department of Applied Mathematics and Theoretical Physics, University of Cambridge, Wilberforce Road, Cambridge, CB3 0WA UK; 3 Department of Chemistry, University of Cambridge, Lensfield Road, Cambridge, CB2 1EW UK; 4 Department of Physics, University of Bath, Bath, BA2 7AY UK

**Keywords:** Nonequilibrium dynamical fluctuations, Large deviations, Microscopic fluctuation theory, Irreversible Markov chains, 82C22, 82C35, 60J27, 60F10

## Abstract

We discuss a canonical structure that provides a unifying description of dynamical large deviations for irreversible finite state Markov chains (continuous time), Onsager theory, and Macroscopic Fluctuation Theory (MFT). For Markov chains, this theory involves a non-linear relation between probability currents and their conjugate forces. Within this framework, we show how the forces can be split into two components, which are orthogonal to each other, in a generalised sense. This splitting allows a decomposition of the pathwise rate function into three terms, which have physical interpretations in terms of dissipation and convergence to equilibrium. Similar decompositions hold for rate functions at level 2 and level 2.5. These results clarify how bounds on entropy production and fluctuation theorems emerge from the underlying dynamical rules. We discuss how these results for Markov chains are related to similar structures within MFT, which describes hydrodynamic limits of such microscopic models.

## Introduction

We consider dynamical fluctuations in systems described by Markov chains. The nature of such fluctuations in physical systems constrains the mathematical models that can be used to describe them. For example, there are well-known relationships between equilibrium physical systems and detailed balance in Markov models [20, Sect. 5.3.4]. Away from equilibrium, fluctuation theorems [12, 19, 25, 32, 37] and associated ideas of local detailed balance [32, 39] have shown how the entropy production of a system must be accounted for correctly when modelling physical systems. However, the mathematical structures that determine the probabilities of non-equilibrium fluctuations are still only partially understood.

We characterise dynamical fluctuations using an approach based on the *Onsager–Machlup (OM) theory* [36], which is concerned with fluctuations of macroscopic properties of physical systems (for example, density or energy). Associated to these fluctuations is a *large-deviation principle* (LDP), which encodes the probability of rare dynamical trajectories. The classical ideas of OM theory have been extended in recent years, through the *Macroscopic Fluctuation Theory* (MFT) of Bertini et al. [7]. This theory uses an LDP to describe path probabilities for the density and current in diffusive systems, on the hydrodynamic scale. At the centre of MFT is a decomposition of the current into two orthogonal terms, one of which is symmetric under time-reversal, and another which is anti-symmetric. The resulting theory is a general framework for the analysis of dynamical fluctuations in a large class of non-equilibrium systems. It also connects dynamical fluctuations with thermodynamic quantities like free energy and entropy production, and with associated non-equilibrium objects like the quasi-potential (which extends the thermodynamic free energy to non-equilibrium settings).

Here, we show how several features that appear in MFT can be attributed to a general structure that characterises dynamical fluctuations in microscopic Markov models. That is, the properties of the hydrodynamic (MFT) theory can be traced back to the properties of the underlying stochastic processes. Our approach builds on recent work by Mielke, Renger and M. A. Peletier, in which the analogue of the OM theory for reversible Markov chains has been described in terms of a *generalised gradient-flow structure* [43]. To describe non-equilibrium processes, that theory must be generalised to include irreversible Markov chains. This can be achieved using the canonical structure of fluctuations discovered by Maes and Netočný [38]. Extending their approach, we decompose currents in the system into two parts, and we identify a kind of orthogonality relationship associated with this decomposition. However, in contrast to the classical OM theory and to MFT, the large deviation principles that appear in our approach have non-quadratic rate functions, which means that fluxes have non-linear dependence on their conjugate forces. Thus, the idea of orthogonality between currents needs to be generalised, just as the notion of gradient flows in macroscopic equilibrium systems can be extended to generalised gradient flows.

The central players in our analysis are the probability density $\rho$ and the probability current *j*. For a given Markov chain, the relation between these quantities is fully encoded in the master equation, which also fully specifies the dynamical fluctuations in that model. However, thermodynamic aspects of the system—the roles of heat, free energy, and entropy production—are not apparent in the master equation. Within the Onsager–Machlup theory, these thermodynamic quantities appear in the action functional for paths, and solutions of the master equation appear as paths of minimal action. Hence, the structure that we discuss here, and particularly the decomposition of the current into two components, links the dynamical properties of the system to thermodynamic concepts, both for equilibrium and non-equilibrium systems.

### Summary

We now sketch the setting considered in this article (precise definitions of the systems of interest and the relevant currents, densities and forces will be given in Sect. 2).

We introduce a large parameter $\mathcal {N}$, which might be the size of the system (as in MFT) or a large number of copies of the system (an ensemble), as considered for Markov chains in [39]. Then let $(\hat{\rho }^{\;\!{\mathcal {N}}}_t,\hat{\jmath }^{\;\!{\mathcal {N}}}_t)_{t\in [0,T]}$ be the (random) path followed by the system’s density and current, in the time interval [0, *T*]. Consider a random initial condition such that $\mathrm {Prob}( \hat{\rho }_0^{\;\!{\mathcal {N}}} \approx \rho ) \asymp \exp [-\mathcal {N} I_0(\rho ) ]$, asymptotically as $\mathcal {N}\rightarrow \infty$, for some rate functional $I_0$. Paths that in addition satisfy a continuity equation $\dot{\rho } + {\text {div}} j =0$ have the asymptotic probability

1 $$\begin{aligned} \mathrm {Prob}\left( \left( \hat{\rho }_t^{\;\!{\mathcal {N}}},\hat{\jmath }_t^{\;\!{\mathcal {N}}}\right) _{t\in [0,T]} \approx (\rho _t, j_t)_{t\in [0,T]}\right) \asymp \exp \left\{ -\mathcal {N} I_{[0,T]}\left( (\rho _t, j_t)_{t\in [0,T]}\right) \right\} \end{aligned}$$

with the *rate functional*

2 $$\begin{aligned} I_{[0,T]}\bigl ((\rho _t, j_t)_{t\in [0,T]}\bigr )= I_0(\rho _0) + \frac{1}{2}\int _0^T \varPhi (\rho _t,j_t, F(\rho _t)) \,{\mathrm {d}}t ; \end{aligned}$$

here $F(\rho _t)$ is a force (see (12) below for the precise definition) and $\varPhi$ is what we call the *generalised OM functional*, which has the general form

3 $$\begin{aligned} \varPhi (\rho ,j,f):= \varPsi (\rho ,j) - j \cdot f + \varPsi ^\star (\rho , f), \end{aligned}$$

where $j\cdot f$ is a dual pairing between a current *j* and a force *f*, while $\varPsi$ and $\varPsi ^\star$ are a pair of functions which satisfy

4 $$\begin{aligned} \varPsi ^\star (\rho ,f) = \sup _j\bigl [ j\cdot f - \varPsi (\rho ,j)\bigr ],\quad \text {and}\quad \varPsi (\rho ,j) = \sup _f\bigl [ j\cdot f - \varPsi ^\star (\rho ,f)\bigr ], \end{aligned}$$

as well as $\varPsi ^\star (\rho ,f)=\varPsi ^\star (\rho ,-f)$ and $\varPsi (\rho ,j)=\varPsi (\rho ,-j)$. Note that (4) means that the two functions satisfy a Legendre duality. Moreover, these two functions $\varPsi$ and $\varPsi ^\star$ are strictly convex in their second arguments. Here and throughout, *f* indicates a force, while *F* is a function whose (density-dependent) value is a force.

The large deviation principle stated in (1) is somewhat abstract: for example, $\hat{\rho }_t^{\;\!{\mathcal {N}}}$ might be defined as a density on a discrete space or on $\mathbb {R}^d$, depending on the system of interest. Specific examples will be given below. In addition, all microscopic parameters of the system (particle hopping rates, diffusion constants, etc.) will enter the (system-dependent) functions $\varPsi$, $\varPsi ^\star$ and *F*.

As a preliminary example, we recall the classical Onsager theory [36], in which one considers *n* currents $j=(j^\alpha )_{\alpha =1}^n$ and a set of conjugate applied forces $F=(F^\alpha )_{\alpha =1}^n$. Examples of currents might be particle flow or heat flow, and the relevant forces might be pressure or temperature gradients. The large parameter ${\mathcal {N}}$ corresponds to the size of a macroscopic system. The theory aims to to describe the typical (average) response of the current *j* to the force *F*, and also the fluctuations of *j*. In this (simplest) case, the density $\rho$ plays no role, so the force *F* has a fixed value in $\mathbb {R}^n$. The dual pairing is simply $j\cdot f = \sum _\alpha j^\alpha f^\alpha$ and $\varPsi$ is given by $\varPsi (\rho ,j)=\frac{1}{2} \sum _{\alpha ,\beta } j^\alpha R^{\alpha \beta } j^\beta$, where *R* is a symmetric $n\times n$ matrix with elements $R^{\alpha \beta }$. The Legendre dual of $\varPsi$ is $\varPsi ^\star (\rho ,f) =\frac{1}{2} \sum _{\alpha ,\beta } f^\alpha L^{\alpha \beta } f^\beta$, where $L=R^{-1}$ is the *Onsager matrix*, whose elements are the linear response coefficients of the system. One sees that $\varPsi$ and $\varPsi ^\star$ can be interpreted as squared norms for currents and forces respectively. Denoting this norm by $\Vert j \Vert ^2_{L^{-1}} := \varPsi (\rho ,j)$, one has

5 $$\begin{aligned} \varPhi (\rho ,j,f) = \Vert j - L f \Vert ^2_{L^{-1}}. \end{aligned}$$

On applying an external force *F*, the response of the current *j* is obtained as the minimum of $\varPhi$, so $j=LF$ (that is, $j^\alpha = \sum _\beta L^{\alpha \beta } F^\beta$). One sees that $\varPhi$ measures the deviation of the current *j* from its expected value *LF*, within an appropriate norm. From the LDP (1), one sees that the size of this deviation determines the probability of observing a current fluctuation of this size.

In this article, we show in Sect. 2 that finite Markov chains have an LDP rate functional of the form (3), where $\varPhi$ (and thus $\varPsi ^\star$) are *not* quadratic. In that case, $\rho$ and *j* correspond to probability densities and probability currents, while the transition rates of the Markov chain determine the functions *F*, $\varPsi$ and $\varPsi ^\star$. Since $\varPsi$ and $\varPsi ^\star$ measure respectively the sizes of the currents and forces, we interpret them as generalisations of the squared norms that appear in the classical case. The resulting $\varPhi$ is not a squared norm, but it is still a non-negative function that measures the deviation of *j* from its most likely value. This leads to nonlinear relations between forces and currents. The MFT theory [7] also fits in this framework, as we show in Sect. 4: in that case $\rho ,j$ are a particle density and a particle current. However, there are relationships between the functions $\varPhi$ for MFT and for general Markov chains, as we discuss in Sect. 4.5.

Hence, the general structure of Eqs. (1)–(4) describes classical OM theory [36], MFT, and finite Markov chains. A benefit is that the terms have a physical interpretation. For a path $(\rho , j)$, the time-reversed path is $(\rho ^*_t,j^*_t):=(\rho _{T-t},-j_{T-t})$. Since both $\varPsi$ and $\varPsi ^\star$ are symmetric in their second argument and thus invariant under time reversal, it holds that $\varPhi (\rho ,j,f) - \varPhi (\rho ^*,j^*,f) = - 2 j \cdot f$. This allows us to identify $j\cdot F(\rho )$ as a rate of entropy production. In contrast, the term $\varPsi (\rho ,j) + \varPsi ^\star (\rho , {F(\rho )})$ is symmetric under time reversal and encodes the frenesy (see [3]). Thus, within this general structure, the physical significance of Eqs. (1)–(4) is that they connect path probabilities to physical notions such as force, current, entropy production and breaking of time-reversal symmetry. Furthermore, we introduce in Sect. 3 decompositions of forces and the (path-wise) rate functional. Sect. 4 shows that some results of MFT originate from generalised orthogonalities of the underlying Markov chains derived in Sect. 3. Similar results hold for time-average large deviation principles, as shown in Sect. 5. In Sect. 6, we show how some properties of MFT can be derived directly from the canonical structure (1)–(4), independent of the specific models of interest. Hence these results of MFT have analogues in Markov chains. Finally we briefly summarise our conclusions in Sect. 7.

## Onsager–Machlup Theory for Markov Chains

In this section, we collect results on forces and currents in Markov chains and on associated LDPs. In particular, we recall the setting of [38, 39]; other references for this section are for example [49] (for the definition of forces and currents in Markov chains) and [43] for LDPs.

### Setting

We consider an irreducible continuous time Markov chain $X_t$ on a finite state space *V* with a unique stationary distribution $\pi$ that satisfies $\pi (x)>0$ for all $x\in V$. The transition rate from state *x* to state *y* is denoted with $r_{xy}$. We assume that $r_{xy}>0$ if and only if $r_{yx}>0$.

We restrict to finite Markov chains for simplicity: the theory can be extended to countable state Markov chains, but this requires some additional assumptions. Briefly, one requires that the Markov chain should be positively recurrent and ergodic (see for instance [9]), for which it is sufficient that (i) the transition rates are not degenerate: $\sum _{y\in V}r_{xy}<\infty$ for all $x\in V$, and (ii) for each $x\in V$, the Markov chain started in *x* almost all trajectories of the Markov chain do not exhibit infinitely many jumps in finite time (“no explosion”). Second, one has to invoke a summability condition for the currents considered below (see, e.g., Eqs. 9 and 10), such that in particular the discrete integration by parts (or summation by parts) formula (15) holds. Finally, note that the cited result for existence and uniqueness of the optimal control potential (the solution to (70)) is only valid for finite state Markov chains.

As usual, we can interpret the state space of the Markov chain as a directed graph with vertices *V* and edges $E=\left\{ xy \bigm | x,y\in V, r_{xy}>0\right\}$, such that $xy\in E$ if and only if $yx\in E$. Let $\rho$ be a probability measure on *V*. We define rescaled transition rates with respect to $\pi$ as

6 $$\begin{aligned} q_{xy}:=\pi (x)r_{xy}, \end{aligned}$$

so that $\rho (x)r_{xy} = \tfrac{\rho (x)}{\pi (x)}q_{xy}$. With this notation, the *detailed balance* condition $\pi (x) r_{xy} = \pi (y) r_{yx}$ reads $q_{xy} = q_{yx}$, so this equality holds precisely if the Markov chain is reversible (i.e. satisfies detailed balance). In general (not assuming reversibility), since $\pi$ is the invariant measure for the Markov chain, one has (for all *x*) that

7 $$\begin{aligned} \sum _y (q_{xy} - q_{yx} ) = 0. \end{aligned}$$

We further define the *free energy* $\mathcal {F}$ on *V* to be the *relative entropy* (or *Kullback–Leibler divergence*) with respect to $\pi$,

8 $$\begin{aligned} \mathcal {F}(\rho ) := \sum _{x} \rho (x) \log \Bigl (\frac{\rho (x)}{\pi (x)}\Bigr ). \end{aligned}$$

The *probability current* $J(\rho )$ is defined as [49, Eq. (7.4)]

9 $$\begin{aligned} J_{xy}(\rho ) := \rho (x)r_{xy}-\rho (y)r_{yx}. \end{aligned}$$

Moreover, for a general current *j* such that $j_{xy}=-j_{yx}$, we define the *divergence* as

10 $$\begin{aligned} {\mathrm{div}\,}j(x) := \sum _{y\in V} j_{xy}. \end{aligned}$$

We say that *j* is *divergence free* if ${\mathrm{div}\,}j(x) = 0$ for every $x \in V$. The time evolution of the probability density $\rho$ is then given by the master equation

11 $$\begin{aligned} \dot{\rho }_t = -{\mathrm{div}\,}J(\rho _t) \end{aligned}$$

(which is often stated as $\dot{\rho }_t = \mathcal {L}^\dag \rho _t$, with the (forward) generator $\mathcal {L}^\dag$).

### Non-linear Flux–Force Relation and the Associated Functionals $\varPsi$ and $\varPsi ^\star$

To apply the theory outlined in Sect. 1.1, the next step is to identify the appropriate forces $F(\rho )$ and also a set of mobilities $a(\rho )$. In this section we define these forces, following [38, 39, 49]. This amounts to a reparameterisation of the rates of the Markov process in terms of physically-relevant variables: an example is given in Sect. 3.5.

To each edge in *E* we assign a *force* *F* and a *mobility* *a*, as

12 $$\begin{aligned} F_{xy}(\rho ) := \log \frac{\rho (x) r_{xy}}{ \rho (y) r_{yx}} \quad \text {and}\quad a_{xy}(\rho ) := 2\sqrt{ \rho (x) r_{xy} \rho (y) r_{yx} }. \end{aligned}$$

Note that $F_{xy}=-F_{yx}$, while $a_{xy}=a_{yx}$: forces have a direction but the mobility is a symmetric property of each edge. The fact that $F_{xy}$ depends on the density $\rho$ means that these forces act in the space of probability distributions. This definition of the force is sometimes also called *affinity* [49, Eq. (7.5)]; see also [1]. With this definition, the probability current (9) is

13 $$\begin{aligned} J_{xy}(\rho ) = a_{xy}(\rho ) \sinh \bigl ( \tfrac{1}{2} F_{xy}(\rho ) \bigr ), \end{aligned}$$

which may be verified directly from the definition $\sinh (x) = (\mathrm{e}^x-\mathrm{e}^{-x})/2$. In contrast to the classical OM theory, this is a *non-linear* relation between forces and fluxes, although one recovers a linear structure for small forces (recall the classical theory in Sect. 1.1, for which $j=Lf$).

Now consider a current *j* defined on *E*, with $j_{xy}=-j_{yx}$, and a general force *f* that satisfies $f_{xy}=-f_{yx}$ (which is not in general given by (12)). Define a dual pair on *E* as

14 $$\begin{aligned} j\cdot f := \frac{1}{2}\sum _{xy} j_{xy}f_{xy}, \end{aligned}$$

where the summation is over all $xy\in E$ (the normalisation 1 / 2 appears because each connected pair of states should be counted only once, but *E* is a set of directed edges, so it contains both *xy* and *yx*, which have the same contribution to $j\cdot f$).

We define the discrete gradient $\nabla g$ by $\nabla ^{x,y}g:=g(y)-g(x)$. The discrete gradient and the divergence defined in (10) satisfy a discrete integration by parts formula: for any function $g:V\rightarrow \mathbb {R}$, since $j_{xy} = -j_{yx}$, we have

15 $$\begin{aligned} -\sum _{x \in V} g(x) {\mathrm{div}\,}j(x) = \frac{1}{2} \sum _{xy} j_{xy} \nabla ^{x,y} g = j\cdot \nabla g. \end{aligned}$$

We will show in Sect. 2.3 that there is an OM functional associated with these forces and currents, which is of the form (3). Since $\varPsi$ and $\varPsi ^\star$ are convex and related by a Legendre transformation, it is sufficient to specify only one of them. The appropriate choice turns out to be

16 $$\begin{aligned} \varPsi ^\star (\rho ,f):=\sum _{xy}a_{xy}(\rho ) \bigl (\cosh \bigl ( \tfrac{1}{2} f_{xy} \bigr )-1\bigr ). \end{aligned}$$

This means that $\varPhi (\rho ,j,f)$ defined in (3) is uniquely minimised for the current $j_{xy} = j^f_{xy}(\rho )$ with

17 $$\begin{aligned} { j^f_{xy}(\rho ) = 2(\delta \varPsi ^\star /\delta f)_{xy} = a_{xy}(\rho ) \sinh (f_{xy}/2), } \end{aligned}$$

as required for consistency with (13). From (4) and (14), one has also

18 $$\begin{aligned} \varPsi (\rho ,j)= \frac{1}{2} \sum _{xy} j_{xy}f^j_{xy}(\rho ) - \sum _{xy} a_{xy}(\rho )\bigl (\cosh \bigl (\tfrac{1}{2} f^j_{xy}(\rho ) \bigr )-1\bigr ), \end{aligned}$$

where

19 $$\begin{aligned} {f^j_{xy}(\rho ):=2{\text {arcsinh}}\left( {j_{xy}/a_{xy}(\rho )}\right) } \end{aligned}$$

is the force required to induce the current *j*.

Physically, $\varPsi ^\star (\rho ,f)$ is a measure of the strength of the force *f* and $\varPsi (\rho ,j)$ is a measure of the magnitude of the current *j*. Consistent with this interpretation, note that $\varPsi$ and $\varPsi ^\star$ are symmetric in their second arguments. Moreover, for small forces and currents, $\varPsi ^\star$ and $\varPsi$ are quadratic in their second arguments, and can be interpreted as generalisations of squared norms of the force and current respectively. Note that Eqs. (16) and (18) can alternatively be represented as

20 $$\begin{aligned} \varPsi (\rho ,j)= \sum _{xy} \biggl [ \frac{1}{2}j_{xy}f^j_{xy}(\rho ) - \sqrt{j_{xy}^2+a_{xy}(\rho )^2} + a_{xy}(\rho )\biggr ] \end{aligned}$$

and

21 $$\begin{aligned} \varPsi ^\star (\rho ,f):=\sum _{xy}\biggl [ \sqrt{j^f_{xy}(\rho )^2+a_{xy}(\rho )^2} - a_{xy}(\rho )\biggr ]. \end{aligned}$$

### Large Deviations and the Onsager–Machlup Functional

As anticipated in Sect. 1.1, the motivation for the definitions of $\varPsi$, $\varPsi ^\star$, and *F* is that there is a large deviation principle for these Markov chains, whose rate function is of the form given in (2). This large deviation principle appears when one considers $\mathcal {N}$ independent copies of the Markov chain.

We denote the *i*th copy of the Markov chain by $X^i_t$ and define the empirical density for this copy as $\hat{\rho }^{\;\!i}_t(x)=\delta _{X^i_t,x}$, where $\delta$ is a Kronecker delta function. Let the times at which the Markov chain $X^i_t$ has jumps in [0, *T*] be $t_1^i, t_2^i, \dots , t^i_{K_i}$. Further denote the state just before the *k*th jump with $x_{k-1}^i$ (such that the state after the *k*th jump is $x_{k}^i$). With this, the empirical current is given by

$$\begin{aligned} \left( \hat{\jmath }_t^{\;\!i}\right) _{xy} = \sum _{k=1}^{K_i} \bigl (\delta _{x,x_{k-1}^i} \delta _{y,x_k^i} - \delta _{y,x_{k-1}^i} \delta _{x,x_{k}^i}\bigr ) \delta \left( t-t_k^i\right) , \end{aligned}$$

where $\delta (t-t_k)$ denotes a Dirac delta. Note that $(\hat{\jmath }_t^{\;\! i})_{xy}=-(\hat{\jmath }_t^{\;\! i})_{yx}$ and the total probability is conserved, as $\sum _x {\mathrm{div}\,}\hat{\jmath }_t^{\;\! i}(x) =0$ (which holds for any discrete vector field with $(\hat{\jmath }_t^{\;\! i})_{xy}=-(\hat{\jmath }_t^{\;\! i})_{yx}$). With a slight abuse of notation we define a similar empirical density and current for the full set of copies as

22 $$\begin{aligned} \hat{\rho }_t^{\;\!\;\!{\mathcal {N}}}:= \frac{1}{{\mathcal {N}}}\sum _{i=1}^{\mathcal {N}}\hat{\rho }^{\;\!i}_t, \quad \text {and}\quad \hat{\jmath }_t^{\;\!\;\!{\mathcal {N}}} := \frac{1}{{\mathcal {N}}}\sum _{i=1}^{\mathcal {N}}\hat{\jmath }^{\;\!i}_t. \end{aligned}$$

Next, we state the large deviation principle where the OM functional appears. For this, we fix a time interval [0, *T*] and consider the large $\mathcal {N}$ limit. We assume that the $\mathcal {N}$ copies at time $t=0$ have initial conditions drawn from the invariant measure of the process (the generalisation to other initial conditions is straightforward). Then, the probability to observe a joint density and current $(\rho _t, j_t)_{t\in [0,T]}$ over the time interval [0, *T*] is in the limit as $\mathcal {N}\rightarrow \infty$ given by (1). That is,

23 $$\begin{aligned} \mathrm {Prob}\Bigl ( \big (\hat{\rho }_t^{\;\!\mathcal {N}},\hat{\jmath }_t^{\;\!\mathcal {N}}\big )_{t\in [0,T]} \approx (\rho _t, j_t)_{t\in [0,T]}\Bigr ) \asymp \exp \bigl \{-\mathcal {N} I_{[0,T]}\bigl ( (\rho _t, j_t)_{t\in [0,T]}\bigr )\bigr \} \end{aligned}$$

with

24 $$\begin{aligned} I_{[0,T]}\bigl ((\rho _t,j_t)_{t\in [0,T]}\bigr ) = {\left\{ \begin{array}{ll} \mathcal {F}(\rho _0) + \frac{1}{2}\int _0^T \varPhi (\rho _t,j_t, F(\rho _t)) \,{\mathrm {d}}t &{} \text {if } \dot{\rho }_t + {\text {div}} j_t = 0\\ +\infty &{} \text {otherwise} \end{array}\right. } \end{aligned}$$

Here, $F(\rho )$ is the force defined in (12) and the condition $\dot{\rho }_t + {\mathrm{div}\,}j_t = 0$ has to hold for almost all $t\in [0,T]$. Moreover, $\varPhi$ is of the form $\varPhi (\rho ,j,f) = \varPsi (\rho ,j) - j\cdot f + \varPsi ^\star (\rho ,f)$ stated in (3), and the relevant functions $\varPsi$, $\varPsi ^\star$ and $\mathcal {F}$ are those of (16), (18) and (8). This LDP was formally derived in [38, 39]. Since the quantities defined in (22) are simple averages over independent copies of the same Markov chain, this LDP may also be proven by direct application of Sanov’s theorem, which provides an interpretation of $I_{[0,T]}$ as a relative entropy between path measures; we sketch the derivation in Appendix A. For finite-state Markov chains, (23) and (24) also follow (by contraction) from [48, Theorem 4.2], which provides a rigorous proof.

We emphasise that the arguments $\rho$ and *j* of the function $\varPhi$ correspond to the random variables that appear in the LDP, while the functions *F*, $\varPsi$ and $\varPsi ^\star$ that appear in $\varPhi$ encapsulate the transition rates of the Markov chain. Thus, by reparameterising the rates $r_{xy}$ in terms of forces *F* and mobilities *a*, we arrive at a representation of the rate function which helps to make its properties transparent (convexity, positivity, symmetries such as (25)).

We note that for reversible Markov chains, the force $F(\rho )$ is a pure gradient $F=\nabla G$ for some potential *G* (see Sect. 3), in which case one may write $j\cdot F=\sum _x \dot{\rho }(x) G(x)$, which follows from an integration by parts and application of the continuity equation. In this case, Mielke, M. A. Peletier, and Renger [43] also identified a slightly different canonical structure to the one presented here, in which the dual pairing is $\sum _x v(x) G(x)$, for a velocity $v(x)=\dot{\rho }(x)$ and a potential *G*. The analogues of $\varPsi$ and $\varPsi ^\star$ in that setting depend on *v* and *G* respectively, instead of *j* and *F*. The setting of (3) and (4) is more general, in that the functions $\varPsi ,\varPsi ^\star$ for the velocity/potential setting are fully determined by those for the current/force setting. Also, focusing on the velocity *v* prevents any analysis of the divergence-free part of the current, and restricting to potential forces does not generalise in a simple way to irreversible Markov chains. For this reason, we use the current/force setting in this work.

In a separate development, Maas [35] identified a quadratic cost function for paths (in fact a metric structure) for which the master equation (11) is the minimiser in the case of reversible dynamics. This metric corresponds to the solution of an optimal mass transfer problem which seems to have no straightforward extension to irreversible systems. Of course, in the reversible case, the pathwise rate function (24) has the same minimiser, but is non-quadratic and therefore does not correspond to a metric structure, so there is no simple geometrical interpretation of (24). It seems that the non-quadratic structure in the rate function is essential in order capture the large deviations encoded by (23).

### Time-Reversal Symmetry, Entropy Production, and the Gallavotti–Cohen Theorem

The rate function for the large-deviation principle (23) is given by (24), which has been written in terms of forces *F*, currents *j*, and densities $\rho$. To explain why it is useful to write the rate function in this way, we compare the probability of a path $(\rho _t,j_t)_{t\in [0,T]}$ with that of its time-reversed counterpart $(\rho _t^*,j_t^*)_{t\in [0,T]}$, where $(\rho ^*_t,j^*_t) =(\rho _{T-t},-j_{T-t})$ as before.

In this case, the fact that $\varPsi$ and $\varPsi ^\star$ are both even in their second argument means that

25 $$\begin{aligned} -\frac{1}{{\mathcal {N}}} \log&\,\frac{ \mathrm {Prob}\Bigl ( \big (\hat{\rho }_t^{\;\!\mathcal {N}},\hat{\jmath }_t^{\;\!\mathcal {N}}\big )_{t\in [0,T]} \approx (\rho _t, j_t)_{t\in [0,T]}\Bigr ) }{\mathrm {Prob}\Bigl ( \big (\hat{\rho }_t^{\;\!\mathcal {N}},\hat{\jmath }_t^{\;\!\mathcal {N}}\big )_{t\in [0,T]} \approx \big (\rho _t^*, j_t^*\big )_{t\in [0,T]}\Bigr )} \nonumber \\&\asymp I_{[0,T]}\Bigl ( (\rho _t, j_t)_{t\in [0,T]}\Bigr ) - I_{[0,T]}\Bigl ( \big (\rho _t^*, j_t^*\big )_{t\in [0,T]}\Bigr )\nonumber \\&= \mathcal {F}(\rho _0) - \mathcal {F}(\rho _T) - \int _0^T j_t\cdot F(\rho _t)\,{\mathrm {d}}t. \end{aligned}$$

This formula is a (finite-time) statement of the Gallavotti–Cohen fluctuation theorem [19, 32]: see also [12, 37]. It also provides a connection to physical properties of the system being modelled, via the theory of stochastic thermodynamics [50]. The terms involving the free energy $\mathcal {F}$ come from the initial conditions of the forward and reverse paths, while the integral of $j\cdot F$ corresponds to the heat transferred from the system to its environment during the trajectory [50, Eqs. (18), (20)]. This latter quantity—which is the time-reversal antisymmetric part of the pathwise rate function—is related (by a factor of the environmental temperature) to the entropy production in the environment [37]. The definition of the force *F* in (12) has been chosen so that the dual pairing $j\cdot F$ is equal to this rate of heat flow: this means that the forces and currents are conjugate variables, just as (for example) pressure and volume are conjugate in equilibrium thermodynamics. See also the example in Sect. 3.5.

## Decomposition of Forces and Rate Functional

We now introduce a splitting of the force $F(\rho )$ into two parts $F^S(\rho )$ and $F^A$, which are related to the behaviour of the system under time-reversal, as well as to the splitting of the heat current into “excess” and “housekeeping” contributions [50]. We use this splitting to decompose the function $\varPhi$ into three pieces, which allows us to compare (for example) the behaviour of reversible and irreversible Markov chains. This splitting also mirrors a similar construction within MFT [7], and this link will be discussed in Sect. 4. Related splittings have been introduced elsewhere; see [30] and [47] for decompositions of forces in stochastic differential equations, and [13] for decompositions of the instantaneous current in interacting particle systems.

### Splitting of the Force According to Time-Reversal Symmetry

We define the *adjoint process* associated with the original Markov chain of interest. The transition rates of the adjoint process are $r^*_{xy}:=\pi (y)r_{yx}\pi (x)^{-1}$. It is easily verified that the adjoint process has invariant measure $\pi$, so $q^*_{xy}:=\pi (x)r^*_{xy}=q_{yx}$. Under the assumption that the initial distribution is sampled from the steady state, the probability to observe a trajectory for the adjoint process coincides with the probability to observe the time-reversed trajectory for the original process.

From the definition of $F(\rho )$ in (12), we can decompose this force as

26 $$\begin{aligned} F_{xy}(\rho ) = F^S_{xy}(\rho ) + F^A_{xy} \end{aligned}$$

with

27 $$\begin{aligned} F^S_{xy}(\rho ) := -\nabla ^{x,y}\log \frac{\rho }{\pi }, \qquad F^A_{xy} := \log \frac{q_{xy}}{q_{yx}}. \end{aligned}$$

With this choice, we note that the equivalent force for the adjoint process

$$\begin{aligned} F^*(\rho ) = \log \frac{\rho (x) r^*_{xy} }{ \rho (y) r^*_{yx} }, \end{aligned}$$

satisfies $F^*(\rho )=F^S(\rho ) - F^A$. So taking the adjoint inverts the sign of $F^A$ (the “antisymmetric” force) but leaves $F^S(\rho )$ unchanged (the “symmetric” force). For a reversible Markov chain, the adjoint process coincides with the original one, and $F^A=0$.

#### Lemma 1

Given $\rho$, with the mobility $a(\rho )$ of (12), the forces $F^S(\rho )$ and $F^A$ satisfy

28 $$\begin{aligned} \sum _{xy}\sinh \Bigl (F^S_{xy}(\rho )/2\Bigr )\;\! a_{xy}(\rho ) \sinh \Bigl (F^A_{xy}/2\Bigr )=0. \end{aligned}$$

#### Proof

From the definitions of $F^S(\rho )$, $F^A$, $a_{xy}$ and $\sinh$, one has

$$\begin{aligned} a_{xy}(\rho )\sinh \Big ( F^S_{xy}(\rho )/2\Big )=\left( \frac{\rho (x)}{\pi (x)}-\frac{\rho (y)}{\pi (y)}\right) \sqrt{q_{xy}q_{yx}} \end{aligned}$$

and $$\sinh (F^A_{xy}/2) = (q_{xy}q_{yx})^{-1/2}(q_{xy}-q_{yx})/2$$. Hence

$$\begin{aligned}&\sum _{xy}\sinh \Bigl (F^S_{xy}(\rho )/2\Bigr )\;\! a_{x,y}(\rho ) \sinh \Bigl (F^A_{xy}/2\Bigr ) = \frac{1}{2} \sum _{xy} \left( \frac{\rho (x)}{\pi (x)}-\frac{\rho (y)}{\pi (y)}\right) (q_{xy}-q_{yx})\\&\quad = \sum _x \frac{\rho (x)}{\pi (x)}\sum _y(q_{xy}-q_{yx})=0, \end{aligned}$$

where the last equality uses (7). This establishes (28). $\square$

In Sect. 4.4, we will reformulate the so-called Hamilton–Jacobi relation of MFT in terms of forces, and show that this yields an equation analogous to (28).

### Physical Interpretation of $F^S$ and $F^A$

In stochastic thermodynamics, one may identify $F^A_{xy}$ as the *housekeeping heat* (or *adiabatic entropy production*) associated with a single transition from state *x* to state *y*, see [16, 50]. (Within the Markov chain formalism, there is some mixing of the notions of force and energy: usually an energy would be a product of a force and a distance but there is no notion of a distance between states of the Markov chain, so forces and energies have the same units in our analysis.) Hence $j\cdot F^A$ is the rate of flow of housekeeping heat into the environment. The meaning of the housekeeping heat is that for irreversible systems, transitions between states involve unavoidable dissipated heat which cannot be transformed into work (this dissipation is required in order to “do the housekeeping”).

To obtain the physical interpretation of $F^S$, we also define

29 $$\begin{aligned} D(\rho ,j) : =\frac{1}{2} \sum _{xy} j_{xy} \log \frac{\rho (y)\pi (x)}{\rho (x)\pi (y)}. \end{aligned}$$

For a general path $(\rho _t,j_t)_{t\in [0,T]}$ that satisfies $\dot{\rho }_t = - {\mathrm{div}\,}j_t$, we also identify

30 $$\begin{aligned} \frac{d}{dt} \mathcal {F}(\rho _t) = \sum _x \dot{\rho }_t(x) \log \frac{\rho _t(x)}{\pi (x)} = \frac{1}{2}\sum _{xy} (j_t)_{xy} \nabla ^{x,y}\log \frac{\rho }{\pi }= D(\rho _t,j_t), \end{aligned}$$

where we used (8), (15). That is, $D(\rho ,j)$ is the change in free energy induced by the current *j*. Moreover it is easy to see that

31 $$\begin{aligned} F^S_{xy}(\rho ) = -\nabla ^{x,y} \frac{\delta \mathcal {F}}{\delta \rho }, \end{aligned}$$

where $\frac{\delta \mathcal {F}}{\delta \rho }$ denotes the functional derivative of the free energy $\mathcal {F}$ given in (8). (Note that the functional derivative $\delta \mathcal {F}/\delta \rho$ is simply $\partial {\mathcal {F}}/\partial \rho$ in this case, since $\rho$ is defined on a discrete space. We retain the functional notation to emphasise the connection to the general setting of Sect. 1.1.) Also, the last identity in (30) can be phrased as

32 $$\begin{aligned} j\cdot F^S(\rho ) = -D(\rho ,j). \end{aligned}$$

The same identity, with an integration by parts, shows that

33 $$\begin{aligned} D(\rho , j) = 0 \text { if }j \text { is divergence free.} \end{aligned}$$

Equation (31) shows that the symmetric force $F^S$ is minus the gradient of the free energy, so the heat flow associated with the dual pairing of *j* and $F^S$ is equal to (the negative of) the rate of change of the free energy. It follows that the right hand side of (25) can alternatively be written as $-\int j\cdot F^A\, \mathrm {d}t$.

We also recall from Sect. 2.2 that the force *F* acts in the space of probability densities: $F_{xy}$ depends not only on the states *x*, *y* but also on the density $\rho$. (Physical forces acting on individual copies of the system should not depend on $\rho$ since each copy evolves independently, but *F* includes entropic terms associated with the ensemble of copies.) To understand this dependence, it is useful to write $\mathcal {F}(\rho ) = -\sum _x \rho (x) \log \pi (x) + \sum _x \rho (x) \log \rho (x)$. We also write the invariant measure in a Gibbs-Boltzmann form: $\pi (x) = \exp (-U(x))/Z$, where *U*(*x*) is the internal energy of state *x* and $Z=\sum _x \exp (-U(x))$ is a normalisation constant. Then $-\sum _x \rho (x) \log \pi (x) = \mathbb {E}_\rho (U) + \log Z$ depends on the mean energy of the system, while $\sum _x \rho (x) \log \rho (x)$ is (the negative of) the mixing entropy, which comes from the many possible permutations of the copies of the system among the states of the Markov chain. From (31) one then sees that $F^S$ has two contributions: one term (independent of $\rho$) that comes from the gradient of the energy *U* and the other (which depends on $\rho$) comes from the gradient of the entropy. These entropic forces account for the fact that a given empirical density $\rho ^{\;\!{\mathcal {N}}}$ can be achieved in many different ways, since individual copies of the system can be permuted among the different states of the system.

### Generalised Orthogonality for Forces

Recalling the definitions of Sect. 3.1, one sees that the current in the adjoint process satisfies an analogue of (13):

34 $$\begin{aligned} J^*_{xy}(\rho ) := a_{xy}(\rho ) \sinh \bigl (\tfrac{1}{2} F^*_{xy}(\rho )\bigr ), \qquad \text {with}\qquad F^*_{xy}(\rho ):= F^S_{xy}(\rho ) - F^A_{xy}. \end{aligned}$$

Comparing with (27), one sees that the adjoint process may also be obtained by inverting $F^A$ (while keeping $F^S(\rho )$ as it is). For $a_{xy}^S(\rho ):= a_{xy}(\rho )\cosh (F^A_{xy}/2)$ the symmetric current is defined as

35 $$\begin{aligned} J^S_{xy}(\rho ) : = a_{xy}^S(\rho )\sinh \bigl ( F^S_{xy}(\rho )/2\bigr ), \end{aligned}$$

which satisfies $J^S_{xy}(\rho ) = (J_{xy}(\rho ) + J^*_{xy}(\rho ))/2$. It is the same for the process and the adjoint process, and also coincides with the current for reversible processes (where $q_{xy}=q_{yx}$, or equivalently $F^A=0$). An analogous formula can also be obtained for the anti-symmetric current. With $a^A_{xy}(\rho ) := a_{xy}(\rho )\cosh (F^S_{xy}(\rho )/2)=a_{xy}(\pi )\bigl (\frac{\rho (x)}{\pi (x)}+\frac{\rho (y)}{\pi (y)}\bigr )/2$, the anti-symmetric current is defined as

36 $$\begin{aligned} J^A_{xy}(\rho ) := a^A_{xy}(\rho )\sinh \bigl (F^A_{xy}/2\bigr ). \end{aligned}$$

It satisfies $$J^A_{xy}(\rho ) = (J_{xy}(\rho ) - J^*_{xy}(\rho ))/2$$.

Let $\varPsi _S^\star$ be the symmetric version of $\varPsi ^\star$ obtained from (16) with $a_{xy}(\rho )$ replaced by $a^S_{xy}(\rho )$. (The Legendre transform of $\varPsi ^\star _S$ is similarly denoted $\varPsi _S$). This leads to a separation of $\varPsi ^\star (\rho ,F(\rho ))$ in a term corresponding to $F^S(\rho )$ and a term corresponding to $F^A$.

#### Lemma 2

The two forces $F^S(\rho )$ and $F^A$ defined in (27) satisfy

37 $$\begin{aligned} \varPsi ^\star (\rho ,F(\rho )) =\varPsi _S^\star \bigl (\rho ,F^S(\rho )\bigr ) + \varPsi ^\star \bigl (\rho ,F^A\bigr ), \end{aligned}$$

#### Proof

Using $\cosh (x+y) = \cosh (x)\cosh (y) + \sinh (x)\sinh (y)$, Lemma 1 and the definition of $a_{xy}^S(\rho )$, we obtain that the left hand side of (37) is given by

38 $$\begin{aligned}&\sum _{xy} a_{xy}(\rho )\bigl (\cosh (F_{xy}(\rho )/2)-1\bigr ) = \sum _{xy} a_{xy}(\rho )\bigl (\cosh (F^S_{xy}(\rho )/2)\cosh \big (F^A_{xy}(\rho )/2\big )-1\bigr )\nonumber \\&\quad = \sum _{xy} a_{xy}^S(\rho )\bigl (\cosh \big (F^S_{xy}(\rho )/2\big ) - 1\bigr ) + \sum _{xy} a_{xy}(\rho )\bigl (\cosh \big (F^A_{xy}(\rho )/2\big ) - 1\bigr ), \end{aligned}$$

which coincides with the right hand side of (37). $\square$

The physical interpretation of Lemma 2 is that the strength of the force $F(\rho )$ can be written as separate contributions from $F^S(\rho )$ and $F^A$. The following corollary allows us to think of a generalised orthogonality of the forces $F^S(\rho )$ and $F^A$.

#### Proposition 3

(Generalised orthogonality) The forces $F^S(\rho )$ and $F^A$ satisfy

39 $$\begin{aligned} \varPsi ^\star \bigl (\rho ,F^S(\rho )+F^A\bigr ) = \varPsi ^\star \bigl (\rho , F^S(\rho )-F^A\bigr ). \end{aligned}$$

#### Proof

This follows directly from Lemma 2 and the symmetry of $\varPsi ^\star (\rho ,\cdot )$. $\square$

We refer to Proposition 3 as a generalised orthogonality between $F^S$ and $F^A$ because $\varPsi ^\star$ is acting as generalisation of a squared norm (see Sect. 1.1), so (39) can be viewed as a nonlinear generalisation of $\Vert F^S + F^A \Vert ^2 = \Vert F^S - F^A \Vert ^2$, which would be a standard orthogonality between forces.

Moreover, Lemma 2 can be used to decompose the OM functional as a sum of three terms.

#### Corollary 4

Let $\varPhi _S$ be defined as in (3) with $(\varPsi ,\varPsi ^\star )$ replaced by $(\varPsi _S,\varPsi _S^\star )$, and $D(\rho ,j)$ as defined in (29). Then

40 $$\begin{aligned} \varPhi (\rho ,j,F(\rho )) =D(\rho ,j) + \varPhi _S\bigl (\rho ,0,F^S(\rho )\bigr ) + \varPhi \bigl (\rho ,j,F^A\bigr ). \end{aligned}$$

#### Proof

We use the definition of $\varPhi$ in (3) and (32) together with Lemma 2 to decompose $\varPhi (\rho ,j,F(\rho ))$ as

41 $$\begin{aligned} \begin{aligned} \varPhi (\rho ,j,F(\rho ))&= D(\rho ,j) +\varPsi _S^\star \bigl (\rho ,F^S(\rho )\bigr ) + \Bigl [ \varPsi (\rho ,j) - j\cdot F^A+\varPsi ^\star \bigl (\rho ,F^A\bigr )\Bigr ] \\&=D(\rho ,j) + \varPhi _S\bigl (\rho ,0,F^S(\rho )\bigr ) + \varPhi \bigl (\rho ,j,F^A\bigr ), \end{aligned} \end{aligned}$$

which proves the claim. $\square$

Recall from Sect. 1.1 that $\varPhi$ measures how much the current *j* deviates from the typical (or most likely) current $J(\rho )$. One sees from (40) that it can be large for three reasons. The first term is large if the current is pushing the system up in free energy (because *D* is the rate of change of free energy induced by the current *j*). The second term comes from the time-reversal symmetric (gradient) force $F^S(\rho )$, which is pushing the system towards equilibrium. The third term comes from the time-reversal anti-symmetric force $F^A$; namely, it measures how far the current *j* is from the value induced by the force $F^A$.

Corollary 4 also makes it apparent that the free energy $\mathcal {F}$ is monotonically decreasing for solutions of (11), which are minimisers of $I_{[0,T]}$.

#### Corollary 5

The free energy $\mathcal {F}$ is monotonically decreasing along minimisers of the rate function $I_{[0,T]}$. Its rate of change is given by

42 $$\begin{aligned} \frac{d}{dt} \mathcal {F}(\rho _t) = -\varPsi ^\star _S\bigl (\rho _t,F^S(\rho _t)\bigr ) - \varPhi \bigl (\rho _t,J(\rho _t),F^A(\rho _t)\bigr ). \end{aligned}$$

#### Proof

For minimisers of the rate function one has $\varPhi =0$. Hence (30) and Corollary 4 imply that

43 $$\begin{aligned} \frac{d}{dt} \mathcal {F}(\rho _t) = D(\rho ,j) = -\varPsi ^\star _S\bigl (\rho _t,F^S(\rho _t)\bigr ) - \varPhi \bigl (\rho _t,J(\rho _t),F^A(\rho _t)\bigr ). \end{aligned}$$

Both $\varPsi ^\star$ and $\varPhi$ are non-negative, so $\mathcal {F}$ is indeed monotonically decreasing. $\square$

### Hamilton–Jacobi Like Equation for Markov Chains

It is also useful to note at this point an additional aspect of the orthogonality relationships presented here, which has connections to MFT (see Sect. 4). We formulate an analogue of the Hamilton–Jacobi equation of MFT, as follows. Define

44 $$\begin{aligned} \mathbb {H}(\rho ,\xi ) = \frac{1}{2}\left[ \varPsi ^\star (\rho ,F(\rho ) + 2\xi ) - \varPsi ^\star (\rho ,F(\rho )) \right] , \end{aligned}$$

which we refer to as an *extended Hamiltonian*, for reasons discussed in Sect. 6.3 (see also Sect. IV.G of [7]).

The *extended Hamilton–Jacobi equation* for a functional $\mathcal {S}$ is then (cf. equation (100) in Sect. 6.3) given by

45 $$\begin{aligned} \mathbb {H}\left( \rho ,\nabla \frac{\delta \mathcal {S}}{\delta \rho }\right) =0. \end{aligned}$$

Note that the free energy $\mathcal {F}$ defined in (8) solves (45), which follows from Proposition 3 (using (31) and that $\varPsi ^\star$ is symmetric in its second argument). In fact (see Proposition 13), the free energy is the maximal solution to this equation. In MFT, the analogous variational principle can be useful, as a characterisation of the invariant measure of the process. Here, one has a similar characterisation of the (non-equilibrium) free energy.

Since (45) with $\mathcal {S} = \mathcal {F}$ provides a characterisation of the free energy $\mathcal {F}$, which is uniquely determined by the invariant measure $\pi$ of the process, it follows that (45) must be equivalent to the condition that $\pi$ satisfies ${\mathrm{div}\,}J(\pi )=0$: recall (11). Writing everything in terms of the rates of the Markov chain and its adjoint, (45) becomes

$$\begin{aligned} \sum _x \rho (x) \sum _{y} [r_{xy} - r^*_{xy}] = 0, \end{aligned}$$

which must hold for all $\rho$: from the definition of $r^*$ one then has $\sum _y \pi (x)r_{xy}=\sum _y \pi (y)r_{yx}$, which is indeed satisfied if and only if $\pi$ is invariant (cf. Eq. (7)).

**Fig. 1:**
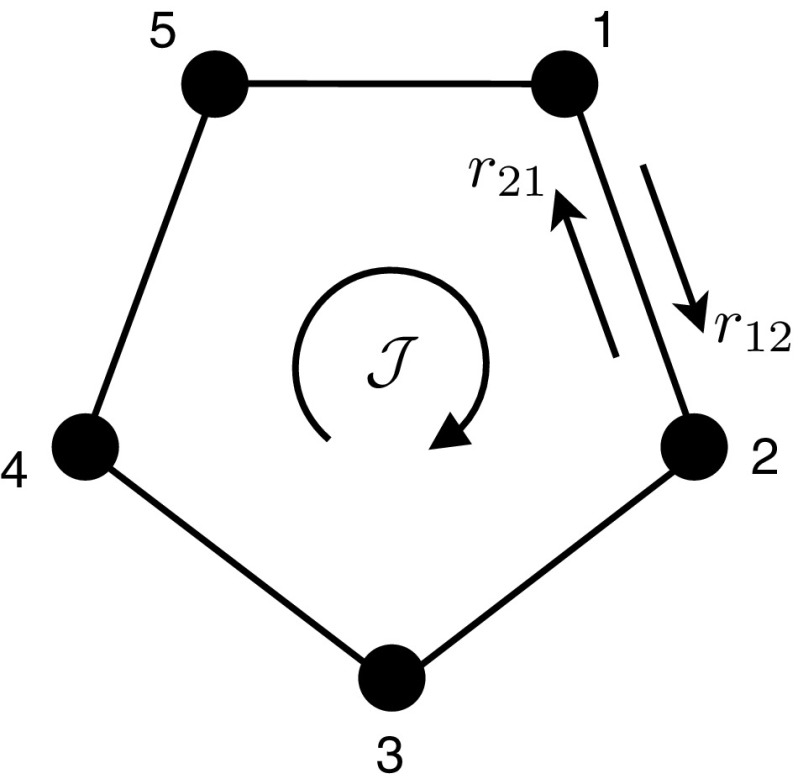
Illustration of a simple Markov chain with $n=5$ states arranged in a circle. The transition rates between states are $r_{i,i\pm 1}$. If the Markov chain is not reversible, there will be a steady-state probability current ${\mathcal {J}}$ corresponding to a net drift of the system around the circle

### Example: Simple Ring Network

To illustrate these abstract ideas, we consider a very simple Markov chain, in which *n* states are arranged in a circle, see Fig. 1. So $V=\{1,2,\dots ,n\}$ and the only allowed transitions take place between state *x* and states $x\pm 1$ (to incorporate the circular geometry we interpret $n+1=1$ and $1-1=n$). In physics, such Markov chains arise (for example) as simple models of nano-machines or motors, where an external energy source might be used to drive circular motion [29, 53]. Alternatively, such a Markov chain might describe a protein molecule that goes through a cyclic sequence of conformations, as it catalyses a chemical reaction [31]. In both cases, the systems evolve stochastically because the relevant objects have sizes on the nano-scale, so thermal fluctuations play an important role.

To apply the analysis presented here, the first step is to identify forces and mobilities, as in (12). Let $R_x = \sqrt{r_{x,x+1} r_{x+1,x}}$. The invariant measure may be identified by solving $\sum _y \pi (x) r_{xy}= \sum _y \pi (y) r_{yx}$ subject to $\sum _y \pi (y)=1$. Finally, one computes the steady state current ${\mathcal {J}} = \pi (x) r_{x,x+1} - \pi (x\!+\!1) r_{x+1,x}$, where the right hand side is independent of *x* (this follows from the steady-state condition on $\pi$). The original Markov process has 2*n* parameters, which are the rates $r_{x,x\pm 1}$: these are completely determined by the $n-1$ independent elements of $\pi$, the *n* mobilities $(R_x)_{x=1}^n$ and the current $\mathcal {J}$. The idea is that this reparameterisation allows access to the physically important quantities in the system.

From the definitions of $\mathcal {J}$ and *R*, it may be verified that

$$\begin{aligned} 2 \pi (x) r_{x,x+1} = \sqrt{{\mathcal {J}}^2 + 4 R_x^2 \pi (x)\pi (x\!+\!1)} + {\mathcal {J}}, \end{aligned}$$

and similarly $2 \pi (x\!+\!1) r_{x+1,x} = \sqrt{{\mathcal {J}}^2 + 4 R_x^2 \pi (x)\pi (x\!+\!1)} - {\mathcal {J}}.$ Then write

46 $$\begin{aligned} \rho (x) r_{x,x+1}= & {} R_x \sqrt{\rho (x)\rho (x+1)} \times \sqrt{ \frac{\rho (x)\pi (x\!+\!1)}{\rho (x\!+\!1)\pi (x)} } \cdot \nonumber \\&\times \left( \frac{\sqrt{{\mathcal {J}}^2 + 4 R_x^2 \pi (x)\pi (x\!+\!1)} + {\mathcal {J}}}{ \sqrt{{\mathcal {J}}^2 + 4 R_x^2 \pi (x)\pi (x\!+\!1)} - {\mathcal {J}} } \right) ^{1/2}. \end{aligned}$$

In this case, we can identify the three terms as

47 $$\begin{aligned} \rho (x) r_{x,x+1} = \frac{1}{2} a_{x,x+1}(\rho ) \times \exp \left( F^S_{x,x+1}(\rho )/2\right) \times \exp \left( F^A_{x,x+1}/2\right) , \end{aligned}$$

which allows us to read off the mobility *a* and the forces $F^S$ and $F^A$. The physical meaning of these quantities may not be obvious from these definitions, but we show in the following that reparameterising the transition rates in this way reveals structure in the dynamical fluctuations.

For example, equilibrium models (with detailed balance) can be identified via $F^A_{x,x+1}=0$ (for all *x*). In general $F^A_{x,x+1}$ is the (steady-state) entropy production associated with a transition from *x* to $x+1$, see Sect. 3.2. The steady state entropy production associated with going once round the circuit is $\sum _x F^A_{x,x+1}=\log \prod _x (r_{x,x+1}/r_{x+1,x})$, as it must be [1].

Now consider the LDP in (23). We consider a large number ($\mathcal {N}$) of identical nano-scale devices, each of which is described by an independent copy of the Markov chain. Typically, each device goes around the circle at random, and the average current is ${\mathcal {J}}$ (so each object performs ${\mathcal {J}}/n$ cycles per unit time). The LDP describes properties of the ensemble of devices. If $\mathcal {N}$ is large and the distribution of devices over states is $\rho$, then the (overwhelmingly likely) time evolution of this distribution is $\dot{\rho } = -{\mathrm{div}\,}J(\rho )$, where the current *J* obeys the simple formula

48 $$\begin{aligned} J_{x,x+1}(\rho ) = a_{x,x+1}(\rho ) \sinh \left( \tfrac{1}{2} \left[ F^S_{x,x+1}(\rho ) + F^A_{x,x+1}\right] \right) , \end{aligned}$$

which is (13), applied to this system. The simplicity of this expression motivates the parametrisation of the transition rates in terms of forces and mobilities. In addition, if one observes some current *j* [not necessarily equal to $J(\rho )$] then the rate of change of free energy of the ensemble can be written compactly as $D(\rho ,j) = -j\cdot F^S(\rho )$, from (32). The quantity $j\cdot F^A$ is the rate of dissipation via housekeeping heat (see Sect. 3.2). This (physically-motivated) splitting of $j\cdot F=j\cdot (F^S+F^A)$ motivates our introduction of the two forces $F^S$ and $F^A$. Note that $j \cdot F$ is the rate of heat flow from the system to its environment, and appears in the fluctuation theorem (25).

Finally we turn to the large deviations of this ensemble of nano-scale objects. There is an LDP (23), whose rate function can be decomposed into three pieces (Corollary 4), because of the generalised orthogonality of the forces $F^S$ and $F^A$ (Lemma 2). This splitting of the rate function is useful because the symmetry properties of the various terms yields bounds on rate functions for some other LDPs obtained from $\varPhi$ by contraction, see Sect. 5.

## Connections to MFT

MFT is a field theory which describes the mass evolution of particle systems in the drift-diffusive regime, on the level of hydrodynamics. In this setting, it can be seen as generalisation of Onsager–Machlup theory [36]. For a comprehensive review, we refer to [7]. This section gives an overview of the theory, focussing on the connections to the results presented in Sects. 2 and 3.

We seek to emphasise two points: first, while the particle currents in MFT and the probability current in Markov chains are very different objects, they both obey large-deviation principles of the form presented in Sect. 1.1. This illustrates the broad applicability of this general setting. Second, we note that many of the particle models for which MFT gives a macroscopic description are Markov chains on discrete spaces. Starting from this observation, we argue in Sect. 4.5 that some results that are well-known in MFT originate from properties of these underlying Markov chains, particularly Proposition 3 and Corollary 4.

### Setting

We consider a large number *N* of indistinguishable particles, moving on a lattice $\varLambda _L$ (indexed by $L\in {\mathbb {N}}$, such that the number of sites $|\varLambda _L|$ is strictly increasing with *L*). These particles are described by a Markov chain, so the relevant forces and currents satisfy the equations derived in Sects. 2 and 3. The hydrodynamic limit is obtained by letting $L\rightarrow \infty$ such that the total density $N/|\varLambda _L|$ converges to a fixed number $\bar{\rho }$. In this limit, the lattice $\varLambda _L$ is rescaled into a domain $\varLambda \subset {\mathbb {R}}^d$ and one can characterise the system by a local (mass) density $\rho :\varLambda \rightarrow [0,\infty )$ together with a local current $j :\varLambda \rightarrow {\mathbb {R}}^d$, which evolve deterministically as a function of time [7, 28]. This time evolution depends on some (density-dependent) applied forces $F(\rho ) :\varLambda \rightarrow {\mathbb {R}}^d$. The force at $x\in \varLambda$ can be written as

49 $$\begin{aligned} { F(\rho )(x)=\hat{f}''(\rho (x))\nabla \rho (x) + E(x),} \end{aligned}$$

where the gradient $\nabla$ denotes a spatial derivative, the function $\hat{f} :[0,\infty )\rightarrow \mathbb {R}$ is a free energy density and $E:\varLambda \rightarrow {\mathbb {R}}^d$ is a drift. (The free energy $\hat{f}$ is conventionally denoted by *f* [7]; here we use a different notation since *f* indicates a force in this work.) With these definitions, the deterministic currents satisfy the linear relation [41]

50 $$\begin{aligned} J(\rho )=\chi (\rho ) F(\rho ), \end{aligned}$$

which is the hydrodynamic analogue of (13). Here, $\chi (\rho ) \in \mathbb {R}^{d \times d}$ is a (density-dependent) mobility matrix.

### Onsager–Machlup Functional

Within MFT, the system is fully specified once the functions $f,\chi ,E$ are given. These three quantities are sufficient to specify both the deterministic evolution of the most likely path $\rho$, and the fluctuations away from it. We can again define an OM functional given by

51 $$\begin{aligned} \varPhi _{{\mathrm {MFT}}}(\rho ,j,f) :=\frac{1}{2}\int _\varLambda \bigl (j-\chi f\bigr )\cdot \chi ^{-1}\bigl (j - \chi f\bigr ) \,{\mathrm {d}}x. \end{aligned}$$

To cast this functional in the form (3), we define the dual pair $\int _\varLambda (j\cdot f) \,{\mathrm {d}}x$, together with the Legendre duals

52 $$\begin{aligned} \varPsi _{{\mathrm {MFT}}}(\rho ,j):=\frac{1}{2}\int _\varLambda j\cdot \chi ^{-1}j \,{\mathrm {d}}x \quad \text {and}\quad \varPsi ^\star _{{\mathrm {MFT}}}(\rho ,f):=\frac{1}{2}\int _\varLambda f\cdot \chi f \,{\mathrm {d}}x. \end{aligned}$$

Given $\rho$ and *f*, we have that $\varPhi _{{\mathrm {MFT}}}$ is uniquely minimised (and equal to zero) for the current $j = \chi (\rho ) f$.

### Large Deviation Principle

Within MFT, one considers an empirical density and an empirical current. We emphasise that these refer to particles, which are interacting and move on the lattice $\varLambda _L$; this is in contrast to the case of Markov chains, where the copies of the system were non-interacting and one considers a density and current of probability. The averaged number of particles at site $i\in \varLambda _L$ is denoted with $\hat{\rho }_t^{\;\!L}(x_i)$, where $x_i$ is the image in the rescaled domain $\varLambda$ of site $i\in \varLambda _L$, and the corresponding particle current is given by $\hat{\jmath }_t^{\;\!L}$ (cf. Sect. VIII.F in [7] for details). Note that both the particle density $\hat{\rho }_t^{\;\!L}$ and the particle current $\hat{\jmath }_t^{\;\!L}$ are random quantities (see also Sect. 4.5).

In keeping with the setting of Sect. 1.1, we focus on paths $(\hat{\rho }_t^{\!\;L},\hat{\jmath }_t^{\;\!L})_{t\in [0,T]}$ in the limit as $L\rightarrow \infty$, where the probability is, analogous to (1), given by

53 $$\begin{aligned} \mathrm {Prob}\Bigl ( \big (\hat{\rho }_t^{L},\hat{\jmath }_t^{\;\!L}\big )_{t\in [0,T]}\approx (\rho _t, j_t)_{t\in [0,T]}\Bigr ) \asymp \exp \bigl \{-|\varLambda _L| I_{[0,T]}^{{\mathrm {MFT}}}\bigl ( (\rho _t, j_t)_{t\in [0,T]}\bigr )\bigr \}. \end{aligned}$$

Note that the parameter ${\mathcal {N}}$ in (1), which is the speed of the LDP, corresponds to the lattice size $|\varLambda _L|$. For the force $F(\rho )$ defined in (49), the rate functional in (53) is given by

54 $$\begin{aligned} I_{[0,T]}^{\mathrm {MFT}}\bigl ( (\rho _t, j_t)_{t\in [0,T]}\bigr )\! =\! {\left\{ \begin{array}{ll} \mathcal {V}(\rho _0)\! +\! \frac{1}{2}\! \int _0^T\!\varPhi _{{\mathrm {MFT}}}(\rho _t,j_t,F(\rho _t)) \,{\mathrm {d}}t &{} \text {if }\dot{\rho }_t\!+\!{\mathrm{div}\,}j_t\! =\! 0\\ +\infty &{} \text {otherwise}. \end{array}\right. } \end{aligned}$$

Here $\mathcal {V}$ is the *quasipotential*, which plays the role of a non-equilibrium free energy. We may think of $\mathcal {V}$ as the macroscopic analogue of the free energy $\mathcal {F}$ defined in (8). It is the rate functional for the process sampled from the invariant measure, which is consistent with the case for Markov chains in (24). We assume that $\mathcal {V}$ has a unique minimiser $\pi$, which is the steady-state density profile (so $\mathcal {V}(\pi )=0$).

An important difference between the Markov chain setting and MFT is that the OM functional for Markov chains is non-quadratic, which is equivalent to a non-linear flux force relation, whereas MFT is restricted to quadratic OM functionals.

Equation (53) is the basic assumption in MFT [7], in the sense that all systems considered by MFT are assumed to satisfy this pathwise LDP. In fact, both the process and its adjoint are assumed to satisfy such LDPs (with similar rate functionals, but different forces) [7].

### Decomposition of the Force *F*

The force *F* in (49) can be written as the sum of a symmetric and an anti-symmetric part, $F(\rho )=F_S(\rho )+F_A(\rho )$, just as in Sect. 3.1. The force for the adjoint process is given by $F^*(\rho )=F_S(\rho )-F_A(\rho )$. Note that, unlike in the case of Markov chains, $F_A(\rho )$ can here depend on $\rho$. More precisely, $F_S(\rho ) = -\nabla \frac{\delta \mathcal {V}}{\delta \rho }$ and $F_A(\rho )$ is given implicitly by $F_A(\rho ) = F(\rho )-F_S(\rho )$.

The symmetric and anti-symmetric currents are defined in terms of the forces $F_S(\rho )$ and $F_A(\rho )$ as $J_S(\rho ) := \chi (\rho )F_S(\rho )$ and $J_A(\rho ) := \chi (\rho )F_A(\rho )$. An important result in MFT is the so-called *Hamilton–Jacobi orthogonality*, which states that

55 $$\begin{aligned} \int _\varLambda J_S(\rho )\cdot \chi (\rho )^{-1} J_A(\rho ) \,{\mathrm {d}}x = 0. \end{aligned}$$

In terms of the forces $F_S(\rho )$ and $F_A(\rho )$, we can restate (55) as

56 $$\begin{aligned} \int _\varLambda F_S(\rho ) \cdot \chi (\rho ) F_A(\rho ) \,{\mathrm {d}}x = 0. \end{aligned}$$

The latter is the quadratic version of the orthogonality (28) of Lemma 1; it is equivalent to

57 $$\begin{aligned}&\int _\varLambda \bigl (F_S(\rho ) + F_A(\rho )\bigr ) \cdot \chi (\rho ) \bigl (F_S(\rho ) + F_A(\rho )\bigr )\,{\mathrm {d}}x\nonumber \\&\quad = \int _\varLambda \bigl (F_S(\rho ) - F_A(\rho )\bigr ) \cdot \chi (\rho ) \bigl (F_S(\rho ) - F_A(\rho )\bigr )\,{\mathrm {d}}x, \end{aligned}$$

or in other words, from (52),

58 $$\begin{aligned} \varPsi ^\star _{{\mathrm {MFT}}}(\rho ,F_S(\rho )+F_A(\rho )) =\varPsi ^\star _{{\mathrm {MFT}}}(\rho ,F_S(\rho )-F_A(\rho )), \end{aligned}$$

which is the result of Proposition 3 in the context of MFT. One can see (39), and hence Proposition 3, as the natural generalisation to the Hamilton–Jacobi orthogonality (55). Again, the MFT describes systems on the macroscopic scale, but the result (58) originates from the result (39), on the microscopic level.

### Relating Markov Chains to MFT: Hydrodynamic Limits

We have discussed a formal analogy between current/density fluctuations in Markov chains and in MFT: the large deviation principles (23) and (53) refer to different objects and different limits, but they both fall within the general setting described in Sect. 1.1. We argue here that the similarities between these two large deviation principles are not coincidental—they arise naturally when MFT is interpreted as a theory for hydrodynamic limits of interacting particle systems.

To avoid confusion between particle densities and probability densities, we introduce (only for this section) a different notation for some properties of discrete Markov chains, which is standard for interacting particle systems. Let $\eta$ represent a state of the Markov chain (in place of the notation *x* of Sect. 2), and let $\mu$ be a probability distribution over these states (in place of the notation $\rho$ of Sect. 2). Let $\jmath$ be the probability current.

We illustrate our argument using the weakly asymmetric simple exclusion process (WASEP) in one dimension, so the lattice is $\varLambda _L=\{1,2,\dots ,L\}$, and each lattice site contains at most one particle, so $V=\{0,1\}^L$. The lattice has periodic boundary conditions and the occupancy of site *i* is $\eta (i)$. Particles hop to the right with rate $L^2$ and to the left with rate $L^2(1-({ E}/L))$, but in either case only if the destination site is empty. Here *E* is a fixed parameter (an external field); the dependence of the hop rates on *L* is chosen to ensure a diffusive hydrodynamic limit (as required for MFT).

The spatial domain relevant for MFT is $\varLambda =[0,1]$: site $i\in \varLambda _L$ corresponds to position $i/L\in \varLambda$. For any probability measure $\mu$ on *V*, one can write a corresponding smoothed particle density $\rho ^\epsilon$ on $\varLambda$, as

59 $$\begin{aligned} \rho ^\epsilon (x) = \frac{1}{L} \sum _{\eta \in V} \sum _{i=1}^L \mu (\eta ) \eta (i/L) \delta ^\epsilon (x-(i/L)), \end{aligned}$$

where $\delta ^\epsilon$ is a smoothed delta function (for example a Gaussian with unit weight and width $\epsilon$, or—more classically—a top-hat function of width $\epsilon$, cf. [28]). Similarly if there is a probability current $\jmath$ in the Markov chain, one can write a smoothed particle current as

60 $$\begin{aligned} j^\epsilon (x) = \frac{1}{L} \sum _{\eta \in V} \sum _{i=1}^L \jmath _{\eta ,\eta ^{i,i+1}} \delta ^\epsilon \left( x-\frac{2i+1}{2L} \right) , \end{aligned}$$

where $\eta ^{i,i+1}$ is the configuration obtained from $\eta$ by moving a particle from site *i* to site $i+1$; if there is no particle on site *i* then define $\eta ^{i,i+1}=\eta$ so that $\jmath _{\eta ,\eta ^{i,i+1}}=0$. Physically, $\rho ^\epsilon$ is the average particle density associated to $\mu$, and $j^\epsilon$ is the particle current associated to $\jmath$.

As noted above, MFT is concerned with the limit $L\rightarrow \infty$. The LDP (23) is not relevant for that limit (it applies when one considers many (${\mathcal {N}}\rightarrow \infty$) independent copies of the Markov chain, with *L* being finite for each copy). However, the rate function $I_{[0,T]}$ that appears in (23) has an alternative physical interpretation, as the relative entropy between two path measures: see Appendix A. This relative entropy can be seen as a property of the WASEP; there is no requirement to invoke many copies of the system. Physically, the relative entropy measures how different is the WASEP from an alternative Markov process with a given probability and current $(\mu _t,\jmath _t)_{t\in [0,T]}$.

The key point is that in cases where MFT applies, one expects that the rate function $I^\mathrm{MFT}_{[0,T]}$ can be related to this relative entropy. In fact, there is a deeper relation between relative entropies and rate functionals: it can be shown that Large Deviation Principles are equivalent to $\varGamma$-convergence of relative entropy functionals (see [42] for details).

Returning to the WASEP, we consider a particle density $(\rho _t,j_t)_{t\in [0,T]}$ that satisfies $\dot{\rho }_t = -{\mathrm{div}\,}j_t$. One then can find (for each *L*) a time-dependent probability and current $(\mu _t^L,\jmath _t^L)_{t\in [0,T]}$, with $\dot{\mu }^L_t = -{\mathrm{div}\,}\jmath ^L_t$, such on taking the limit $\epsilon \rightarrow 0$ *after* $L\rightarrow \infty$, the associated particle densities $(\rho ^\epsilon _t,j^\epsilon _t) \rightarrow (\rho _t,j_t)$ and moreover

61 $$\begin{aligned} \lim _{L\rightarrow \infty }\frac{1}{|\varLambda _L|} I_{[0,T]}\bigl ( \big (\mu _t^L, \jmath _t^L\big )_{t\in [0,T]}\bigr ) = I_{[0,T]}^{\mathrm {MFT}} \bigl ( (\rho _t, j_t)_{t\in [0,T]}\bigr ). \end{aligned}$$

In order to find $(\mu _t^L,\jmath _t^L)_{t\in [0,T]}$, one defines a “controlled” WASEP (similar to (69) in Sect. 5.3), in which the particle hop rates depend on position and time, such that the particle density in the hydrodynamic limit obeys $\dot{\rho }_t = -{\mathrm{div}\,}j_t$.

For interacting particle systems, this “controlled” process is usually obtained by adding a time dependent external field to the system that acts on the individual particles. This was first derived for the symmetric SEP in [27] (see also [4] for a treatment of the zero-range process). For the WASEP (in a slightly different situation with open boundaries) a proof of (61) can e.g. be found in [6], Lemma 3.7.

Moreover, on decomposing $I^\mathrm{MFT}_{[0,T]}$ and $I_{[0,T]}$ as in (3), the separate functions $\varPsi$ and $\varPsi ^\star$ obey formulae analogous to (61): this is the sense in which the structure of the MFT rate function is inherited from the relative entropy of the Markov chains. The quadratic functions $\varPsi$ and $\varPsi ^\star$ in MFT arise because the forces that appear in the underlying Markov chains are small (compared to unity), so second order Taylor expansions of $\varPsi ^\star$ and $\varPsi$ give in the limit the accurate description, similar to [2]. We will return to this discussion in a later publication.

## LDPs for Time-Averaged Quantities

So far we have considered large deviation principles for hydrodynamic limits, and for systems consisting of many independent copies of a single Markov chain. We now show how some of the results derived in Sects. 2 and 3 also have analogues for large deviations for a single Markov chain, in the large-time limit.

### Large Deviations at Level 2.5

Analogous to (22), we define the time averaged empirical measure of a single copy of the Markov chain $\hat{\rho }_{[0,T]}$ and the time averaged empirical current $\hat{\jmath }_{[0,T]}$ as

62 $$\begin{aligned} \hat{\rho }_{[0,T]}:=\frac{1}{T} \int _0^T \hat{\rho }_t \,{\mathrm {d}}t \quad \text {and}\quad \hat{\jmath }_{[0,T]}:=\frac{1}{T}\int _0^T\hat{\jmath }_t \,{\mathrm {d}}t \end{aligned}$$

(where we choose $\hat{\rho }_t=\hat{\rho }_t^1$ and $\hat{\jmath }_t = \hat{\jmath }_t^1$ for the empirical density and current of the single Markov chain, as defined above in Sect. 2.3). For countable state Markov chains, the quantity $(\hat{\rho }_{[0,T]},\hat{\jmath }_{[0,T]})$ satisfies a LDP as $T\rightarrow \infty$:

63 $$\begin{aligned} \mathrm {Prob}\bigl (\big (\hat{\rho }_{[0,T]}, \hat{\jmath }_{[0,T]}\big ) \approx (\rho ,j)\bigr ) \asymp \exp \bigl \{-T I_{2.5}(\rho ,j)\bigr \}. \end{aligned}$$

We refer to such principles as *level 2.5 LDPs*. For countable state Markov chains the rate functional $I_{2.5}(\rho ,j)$ was derived in [39], and was proven rigorously in [8, 9] for Markov chains in the setting of Sect. 2.1 under some additional conditions (see [8, 9] for the details). We can recast the rate functional (see [8, Theorem 6.1]) as

64 $$\begin{aligned} I_{2.5}(\rho ,j) = {\left\{ \begin{array}{ll} \frac{1}{2} \varPhi (\rho ,j,F(\rho )) &{} \text {if }{\mathrm{div}\,}j = 0\\ +\infty &{} \text {otherwise} \end{array}\right. }, \end{aligned}$$

with $\varPhi$ again given by (3), together with (14), (16) and (18).

We have stated this LDP for joint fluctuations of the density and the current. For Markov chains, the LDP for the density and the *flow* is also known as a level-2.5 LDP [9], so our general use of the name level-2.5 for (63) may be non-standard, but it seems reasonable. The rate functional for the density and the current in (63) can be obtained by contraction from the rate functional for the density and the flow (see Theorem 6.1 in [8]).

Using the splitting obtained in Sect. 3.3, we obtain the following representation for the rate functional on level-2.5.

#### Proposition 6

Let *j* be divergence free. Then the level-2.5 rate functional (64) is given by

65 $$\begin{aligned} I_{2.5}(\rho ,j) =\frac{1}{2}\Bigl [\varPhi _S\bigl (\rho ,0,F^S(\rho )\bigr ) + \varPhi \bigl (\rho ,j,F^A\bigr )\Bigr ]. \end{aligned}$$

#### Proof

We note from (33) that $D(\rho ,j)$ vanishes for divergence free currents *j*. The result then directly follows from Corollary 4. $\square$

### Large Deviations for Currents

Proposition 6 is connected to recently-derived bounds on rate functions for currents, see [22, 23, 45, 46]. Indeed, the rate function for current fluctuations can be obtained by contraction from level-2.5, as

66 $$\begin{aligned} { I_\mathrm{current}(j)}:=\inf _\rho I_{2.5}(\rho ,j). \end{aligned}$$

Then, following [23, 46], it may be shown that for any $\rho ,j,f$ one has for $\varPhi$ as in (3) with (14), (16)–(18) that

67 $$\begin{aligned} \varPhi \bigl (\rho ,j,f\bigr ) \le \sum _{xy} \left( j_{xy}-j^{f}_{xy}(\rho )\right) ^2 b_{xy}(\rho ,f) \end{aligned}$$

with $b_{xy}(\rho ,f)=f_{xy}/(4j^f_{xy}(\rho ))$ if $f_{xy}\ne 0$; otherwise $b_{xy}$ is continuously extended by taking $b_{xy}(\rho ,f)=1/(2a_{xy}(\rho ))$. Hence one has the result of [22], that the curvature of the rate function is controlled by the housekeeping heat $F^A$, as

68 $$\begin{aligned} { I_\mathrm{current}(j)}\le I_{2.5}(\pi ,j) = \frac{1}{2} \varPhi \bigl (\pi ,j,F^A\bigr ) \le \frac{1}{2} \sum _{xy} \frac{\bigl (j_{xy}-J_{xy}^\mathrm{ss}\bigr )^2}{4(J_{xy}^\mathrm{ss})^2} J_{xy}^\mathrm{ss} F^A_{xy}, \end{aligned}$$

where $J^\mathrm{ss}:=J(\pi )$ is the steady state current (recall (9)), and the ratio $F^A_{xy}/J^{{\mathrm {ss}}}_{xy}$ must again be interpreted as $2/a_{xy}(\rho )$ in the case where $F^A_{xy}$ (and hence $J^{{\mathrm {ss}}}_{xy}$) vanish. The first step in (68) comes from (66), the second step uses (65) as well as $\varPhi (\pi ,0,F^S)=0$, and the third uses (67).

The significance of the splitting (65) for this result is that $J^\mathrm{ss}_{xy}F_{xy}^A$ is the rate of flow of housekeeping heat associated with edge *xy*: the appearance of the housekeeping heat is natural since the bound comes from the second term in (65), which is independent of $F^S$ and depends only on $F^A$.

### Optimal Control Theory

It will be useful to introduce ideas of optimal control theory, whose relationship with large deviation theory is discussed in [10, 11, 18, 24]. In parallel with our given transition rates $r_{xy}$ we introduce a new process, the *controlled process*, where the rates are modified by a *control potential* $\varphi$, as

69 $$\begin{aligned} \tilde{r}_{xy} := r_{xy} \exp ((\varphi (y)-\varphi (x))/2). \end{aligned}$$

For a given probability distribution $\rho$, we seek a potential $\varphi$ such that the controlled process has invariant measure $\tilde{\pi }:= \rho$. For this we need

$$\begin{aligned} \sum _y \left[ \rho _x r_{xy} \exp ((\varphi (y)-\varphi (x))/2) - \rho _y r_{yx} \exp ((\varphi (x)-\varphi (y))/2)\right] = 0, \end{aligned}$$

or equivalently

70 $$\begin{aligned} {\mathrm{div}\,}j^{F+\nabla \varphi }(\rho ) = \sum _y a_{xy}(\rho ) \sinh \left( (F_{xy}(\rho ) + \nabla ^{x,y}\varphi )/2\right) = 0. \end{aligned}$$

We stress that, for any fixed $\rho$, (70) is equivalent to solving the minimisation problem

71 $$\begin{aligned} \inf _{{\text {div}} j =0}\varPhi \bigl (\rho ,j,F(\rho )\bigr ), \end{aligned}$$

which is also equivalent to maximisation of the Donsker–Varadhan functional, see for example Chapter IV.4 in [15]. A proof for the existence and uniqueness of $\varphi$ can, e.g., be found in [40]. Now assume that $\varphi$ solves (70). The resulting controlled process depends on $\rho$ and has rates $\tilde{r}$ given by (69). Throughout this section, we use tildes to indicate properties of the controlled process: all these quantities depend implicitly on the fixed probability $\rho$. Hence the (time-dependent) measure of the controlled process is $\tilde{\rho }$.

Repeating the analysis of Sect. 2.1 and noting that $\tilde{r}_{xy}\tilde{r}_{yx}=r_{xy}r_{yx}$, we find that $\tilde{a}_{xy}(\tilde{\rho }) := 2\sqrt{\tilde{\rho }(x)\tilde{r}_{xy}\tilde{\rho }(y)\tilde{r}_{yx}} = a_{xy}(\tilde{\rho })$. Also, the force for the controlled process is

72 $$\begin{aligned} \tilde{F}(\tilde{\rho }) = F(\tilde{\rho }) + \nabla \varphi , \end{aligned}$$

which may be decomposed as

73 $$\begin{aligned} \begin{aligned} \tilde{F}^S(\tilde{\rho }) \;\!&:= F^S(\tilde{\rho }) + \nabla \log \frac{\rho }{\pi }=-\nabla \log \frac{\tilde{\rho }}{\rho },\\ \tilde{F}^A \;\!&:= F(\rho )+\nabla \varphi = F^A - \nabla \log \frac{\rho }{\pi }+ \nabla \varphi . \end{aligned} \end{aligned}$$

Thus, the symmetric force in the controlled process vanishes when $\tilde{\rho }= \rho$. The antisymmetric force $\tilde{F}^A$ represents the force observed in the new non-equilibrium steady state $\rho$. If the original process is reversible, then $\varphi =\log \frac{\rho }{\pi }$ so $\tilde{F}^A=F^A=0$.

It is useful to define $\tilde{J}_{xy}(\tilde{\rho }) := a_{xy}(\tilde{\rho }) \sinh (\tilde{F}_{xy}(\tilde{\rho })/2)$ and to identify the steady-state current for the controlled process as

74 $$\begin{aligned} \tilde{J}^{{\mathrm {ss}}} := \tilde{J}(\rho ). \end{aligned}$$

### Decomposition of Rate Functions

The ideas of optimal control theory are useful since they facilitate the further decomposition of the level-2.5 rate function into several contributions.

#### Lemma 7

Suppose that $\rho$ and *j* are given and that ${\mathrm{div}\,}j=0$. Then

75 $$\begin{aligned} I_{2.5}(\rho ,j) = \frac{1}{2} \Bigl [ \varPhi \bigl ( \rho , \tilde{J}^{{\mathrm {ss}}}, F(\rho )\bigr ) + \varPhi \bigl ( \rho , j, \tilde{F}^A \bigr ) \Bigr ], \end{aligned}$$

where $\tilde{J}^\mathrm{ss}$ is given by (74), evaluated in the optimally controlled process whose steady state is $\rho$.

#### Proof

We write

76 $$\begin{aligned} 2I_{2.5}(\rho ,j)&= \varPsi (\rho ,j) - j\cdot F(\rho ) + \varPsi ^\star \bigl (\rho ,F(\rho )\bigr )\nonumber \\&= [\varPsi (\rho ,j) - j\cdot \tilde{F}(\rho ) + \varPsi ^\star ( \rho ,\tilde{F}(\rho )) ]\nonumber \\&\quad + \varPsi ^\star (\rho ,F(\rho )) - \varPsi ^\star ( \rho ,\tilde{F}(\rho )) - j\cdot (F(\rho )-\tilde{F}(\rho ))\nonumber \\&= \varPhi \bigl (\rho , j, \tilde{F}(\rho ) \bigr ) + \varPsi ^\star (\rho ,F(\rho )) - \varPsi ^\star ( \rho ,\tilde{F}(\rho )) + j\cdot \nabla \varphi \end{aligned}$$

where the first line is (3) and (64); the second line is simple rewriting; and the third uses the definition of $\varPhi$ in (3) and also (72) with $\tilde{\rho }=\rho$.

The current $\tilde{J}(\rho )$ satisfies $\varPhi (\rho ,\tilde{J}(\rho ),\tilde{F}(\rho ))=0$ so one has (by definition of $\varPhi$) that $\varPsi ^\star ( \rho ,\tilde{F}(\rho ))=\tilde{J}(\rho )\cdot \tilde{F}(\rho )-\varPsi (\rho ,\tilde{J}(\rho ))$. Using this relation together with (72) and (76), one has

77 $$\begin{aligned} 2I_{2.5}(\rho ,j)= & {} \varPhi \bigl (\rho , j, \tilde{F}(\rho ) \bigr ) + \varPsi ^\star (\rho ,F(\rho )) - \tilde{J}(\rho )\cdot F(\rho )\nonumber \\&+\varPsi (\rho ,\tilde{J}(\rho )) -\tilde{J}(\rho )\cdot \nabla \varphi + j\cdot \nabla \varphi . \end{aligned}$$

Finally we note that ${\mathrm{div}\,}\tilde{J}(\rho )=0$ (since $\rho$ is the invariant measure for the controlled process) and ${\mathrm{div}\,}j=0$ (by assumption), so integration by parts yields $\tilde{J}(\rho )\cdot \nabla \varphi =0=j\cdot \nabla \varphi$; using once more the definition of $\varPhi$ yields (82). $\square$

The physical interpretation of (75) is as follows. The contribution $\frac{1}{2}\varPhi ( \rho , j, \tilde{F}^A )$ is a rate functional for observing an empirical current *j* in the controlled process, while $\frac{1}{2}\varPhi ( \rho , \tilde{J}^{{\mathrm {ss}}}, F(\rho ) )$ is the rate functional for observing an empirical current $\tilde{J}^{{\mathrm {ss}}}$ in the original process. Since $\tilde{J}^{{\mathrm {ss}}}$ is the (deterministic) probability current for the controlled process, one has that the more the controlled process differs from the original one, the larger will be $\varPhi ( \rho , \tilde{J}^{{\mathrm {ss}}}, F(\rho ) )$. Hence the level-2.5 rate functional is large if the controlled process is very different from the original one, as one might expect. The rate functional also takes larger values if the empirical current *j* is very different from the probability current of the controlled process.

We obtain our final representation for the level-2.5 rate functional, consisting of the sum of three different OM functionals.

#### Proposition 8

Let *j* be divergence free. We can represent the level-2.5 rate functional (64) as

78 $$\begin{aligned} I_{2.5}(\rho ,j) = \frac{1}{2} \left[ \varPhi _S\bigl ( \rho , 0, F^S(\rho ) \bigr ) + \varPhi \bigl (\rho ,\tilde{J}^{{\mathrm {ss}}}, F^A \bigr ) + \varPhi \bigl ( \rho , j, \tilde{F}^A \bigr ) \right] . \end{aligned}$$

#### Proof

This follows immediately from Lemma 7 followed by an application of Corollary 4 to $\varPhi \bigl (\rho ,\tilde{J}^{{\mathrm {ss}}}, F^A \bigr )$ and that $D=0$, from (33). $\square$

The three terms in (78) also appear in Lemma 7 and Corollary 4, and their interpretations have been discussed in the context of those results. Briefly, we recall that $I_{2.5}(\rho ,j)$ sets the probability of fluctuations in which a non-typical density $\rho$ and current *j* are sustained over a long time period. The first term in (78) reflects the fact that the free-energy gradient $F^S(\rho )$ tends to push $\rho$ towards the steady state $\pi$, so maintaining any non-typical density is unlikely if $F^S(\rho )$ is large. Similarly, the second term in (78) reflects the fact that large non-gradient forces $F^A$ also tend to suppress the probability that $\rho$ maintains its non-typical value. The final term is the only place in which the (divergence-free) current *j* appears: it vanishes if the current *j* is typical within the controlled process (see Corollary 9); otherwise it reflects the probability cost of maintaining a non-typical circulating current.

### Large Deviations at Level 2

As well the LDP (63), we also consider an (apparently) simpler object, called a *level-2 LDP*, where one considers the density only. It is formally given by

79 $$\begin{aligned} \mathrm {Prob}\left( \hat{\rho }_T \approx \rho \right) \asymp \exp (-T I_{2}(\rho )). \end{aligned}$$

The contraction principle for LDPs [52, Sect. 3.6] states that

80 $$\begin{aligned} I_2(\rho ) = \inf _{j \;\!:\;\! {\mathrm{div}\,}j=0} I_{2.5}(\rho ,j). \end{aligned}$$

Equation (75) is uniquely minimised in its second argument for the divergence free current $j^{\tilde{F}^A}$, such that the contraction over all divergence-free vector fields *j* yields the level-2 rate functional

81 $$\begin{aligned} I_{2}(\rho ) = \frac{1}{2} \varPhi \bigl ( \rho , \tilde{J}^{{\mathrm {ss}}}, F(\rho ) \bigr ). \end{aligned}$$

The same splitting as above finally allows us to write the level 2 rate functional as follows.

#### Corollary 9

The level-2 rate functional can be written as the sum

82 $$\begin{aligned} I_{2}(\rho ) = \frac{1}{2} \Bigl [\varPhi _S\bigl ( \rho , 0, F^S(\rho ) \bigr ) + \varPhi \bigl (\rho ,\tilde{J}^{{\mathrm {ss}}}, F^A\bigr )\Bigr ]. \end{aligned}$$

#### Proof

This follows from (80) and (78), since $\varPhi \bigl ( \rho , j, \tilde{F}^A \bigr )$ has a minimal value of zero. $\square$

This last identity extends the results obtained in [26] on the accelerated convergence to equilibrium for irreversible processes using LDPs from the macroscopic scale (i.e. in the regime of MFT) to Markov chains. The level-2 rate function in (82) can be interpreted as a rate of convergence to the steady state. It was shown in [26] that the rate is higher for irreversible processes, as opposed to reversible ones (as the second term $\varPhi (\rho ,\tilde{J}^{{\mathrm {ss}}}, F^A)=0$ for reversible processes). We remark that splitting techniques for irreversible jump processes have been used to devise efficient MCMC samplers; see for example [5, 34].

### Connection to MFT

Under the assumption that no dynamical phase transition takes place, the time averaged density $\hat{\rho }_{[0,T]}^{L}:=\frac{1}{T}\int _0^T\hat{\rho }_t^{L} \,{\mathrm {d}}t$ and current $\hat{\jmath }_{[0,T]}^{\;\!L}:=\frac{1}{T}\int _0^T\hat{\jmath }_t^{\;\!L}\,{\mathrm {d}}t$ in MFT (recall Sect. 4.3 for definitions) also satisfy a joint LDP in the limit $L,T\rightarrow \infty$: one takes first $L\rightarrow \infty$ and then $T\rightarrow \infty$, see [26, Eq. (36)]. The LDP is similar to (63):

83 $$\begin{aligned} \mathrm {Prob}\left( \left( \hat{\rho }_{[0,T]}^L, \hat{\jmath }_{[0,T]}^L\right) \approx (\rho ,j)\right) \asymp \exp \left\{ -T |\varLambda _L| I_\mathrm{joint}^{{\mathrm {MFT}}}(\rho ,j)\right\} , \end{aligned}$$

where the rate function is, for a density profile $\rho$ and a current *j* with ${\text {div}}j=0$, given by

84 $$\begin{aligned} I_\mathrm{joint}^{{\mathrm {MFT}}}(\rho ,j) = \frac{1}{2} \varPhi _{\mathrm {MFT}}(\rho ,j,F(\rho )). \end{aligned}$$

As for Markov chains (see Sect. 5.1) $I_\mathrm{joint}^{{\mathrm {MFT}}}(\rho ,j) =\infty$ if *j* is not divergence free. If ${\mathrm{div}\,}j=0$ then the rate function can be written in the form [26]

85 $$\begin{aligned}&{ I_\mathrm{joint}^{{\mathrm {MFT}}}(\rho ,j)} =\frac{1}{4} \int _\varLambda \nabla \frac{\delta \mathcal {V}}{\delta \rho }\cdot \chi \nabla \frac{\delta \mathcal {V}}{\delta \rho } \,{\mathrm {d}}x + \frac{1}{4}\int _\varLambda \nabla \varphi \cdot \chi \nabla \varphi \,{\mathrm {d}}x \nonumber \\&\quad + \frac{1}{4} \int _\varLambda (J_F-j)\cdot \chi ^{-1} (J_F-j) \,{\mathrm {d}}x,\quad \end{aligned}$$

such that a contraction to to the density only yields

86 $$\begin{aligned} { I_\mathrm{density}^{{\mathrm {MFT}}}(\rho )} = \frac{1}{4}\int _\varLambda \nabla \frac{\delta \mathcal {V}}{\delta \rho }\cdot \chi \nabla \frac{\delta \mathcal {V}}{\delta \rho } \,{\mathrm {d}}x + \frac{1}{4}\int _\varLambda \nabla \varphi \cdot \chi \nabla \varphi \,{\mathrm {d}}x. \end{aligned}$$

The function $\varphi$ in (85) and (86) is obtained by solving

87 $$\begin{aligned} {\mathrm{div}\,}J_F(\rho ) = 0, \qquad J_F(\rho ) := \chi \nabla \varphi + J_A(\rho ). \end{aligned}$$

Clearly the solution $\varphi$ depends on $\rho$. In essence, we have reduced the minimisation problem (80) to the solution of this PDE. Comparing with (78), we identify the terms $J_F = \chi \tilde{F}^A$ in the MFT setting, and also $\tilde{J}^{{\mathrm {ss}}} = \chi \tilde{F}^A$, so $(\tilde{J}^{{\mathrm {ss}}}-\chi F^A(\rho )) = \chi \nabla \varphi$. We obtain the following representations for (85) and (86) reminiscent of Proposition 8 and Corollary 9.

#### Proposition 10

The rate functional for the joint density and current in MFT, which is given by (85), can be written in terms of the OM functional (51) as

88 $$\begin{aligned} { I_\mathrm{joint}^{{\mathrm {MFT}}}(\rho ,j)} =\frac{1}{2}\Bigl [\varPhi _{\mathrm {MFT}}(\rho ,0,F^S(\rho )) + \varPhi _{\mathrm {MFT}}(\rho ,\tilde{J}^{{\mathrm {ss}}},F^A(\rho )) + \varPhi _{\mathrm {MFT}}(\rho ,j,\tilde{F}^A)\Bigr ], \end{aligned}$$

and (86), the rate functional for the density in MFT, is given by

89 $$\begin{aligned} { I_\mathrm{density}^{{\mathrm {MFT}}}(\rho )} =\frac{1}{2}\Bigl [\varPhi _{\mathrm {MFT}}(\rho ,0,F^S(\rho )) + \varPhi _{\mathrm {MFT}}(\rho ,\tilde{J}^{{\mathrm {ss}}},F^A(\rho ))\Bigr ]. \end{aligned}$$

This proposition is equivalent to Proposition 5 of [26], but has now been rewritten in the language of optimal control theory. As discussed in [26], Eq. (89) quantifies the extent to which breaking detailed balance accelerates convergence of systems to equilibrium, at the hydrodynamic level. For this work, the key point is that this result originates from Corollary 9, which is the equivalent statement for Markov chains (without taking any hydrodynamic limit).

## Consequences of the Structure of the OM Functional $\varPhi$

We have shown that the rate functions for several LDPs in several different contexts depend on functionals $\varPhi$ with the general structure presented in (3) and (4). In this section, we show how this structure alone is sufficient to establish some features that are well-known in MFT. This means that these results within MFT have analogues for Markov chains. Our derivations mostly follow the standard MFT routes [7], but we use a more abstract notation to emphasise the minimal assumptions that are required.

### Assumptions

The following minimal assumptions are easily verified for Markov chains; they are also either assumed or easily proven for MFT. The results of this section are therefore valid in both settings.

We consider a process described by a time-dependent density $\rho$ and current *j*, with an associated continuity equation $\dot{\rho } = -{\mathrm{div}\,}j$ and unique steady state $\pi$. We are given a set of ($\rho$-dependent) forces denoted by $F(\rho )$, a dual pairing $j\cdot f$ between forces and currents, and a function $\varPsi (\rho ,j)$ which is convex in *j* and satisfies $\varPsi (\rho ,j)=\varPsi (\rho ,-j)$. With these choices, the functions $\varPsi ^\star$ and $\varPhi$ are fully specified via (3) and (4). We assume that for initial conditions chosen from the invariant measure, the system satisfies an LDP of the form (1) with rate function of the form (2).

We define an adjoint process for which the probability of a path $(\rho _t,j_t)_{t\in [0,T]}$ is equal to the probability of the time-reversed path $(\rho ^*_t,j^*_t)_{t\in [0,T]}$ in the original process. As above, we define $(\rho ^*_t,j^*_t)=(\rho _{T-t},-j_{T-t})$. We assume that the adjoint process also satisfies an LDP of the form (1), with rate function $I^*_{[0,T]}$. Hence we must have

90 $$\begin{aligned} I^*_{[0,T]}\bigl ((\rho _t, j_t)_{t\in [0,T]}\bigr ) = I_{[0,T]}\bigl (\big (\rho ^*_t, j^*_t\big )_{t\in [0,T]}\bigr ). \end{aligned}$$

Moreover, we assume that $I^*_{[0,T]}$ may be obtained from *I* by replacing the force $F(\rho )$ with some adjoint force $F^*(\rho )$. That is,

91 $$\begin{aligned} I^*_{[0,T]}\bigl ((\rho _t, j_t)_{t\in [0,T]}\bigr )= I_0(\rho _0) + \frac{1}{2}\int _0^T \varPhi (\rho _t,j_t, F^*(\rho _t)) \,{\mathrm {d}}t. \end{aligned}$$

Here, $I_0$ is the rate function associated with fluctuations of the density $\rho$, for a system in its steady state. That is, within the steady state, $\mathrm {Prob}(\hat{\rho }^{\;\!{\mathcal {N}}}\approx \rho ) \asymp \exp (-{\mathcal {N}}I_0(\rho ))$. For Markov chains, $I_0=\mathcal {F}$, the free energy; for MFT we have $I_0=\mathcal {V}$, the quasipotential. In the following we refer to $I_0$ as the free energy.

### Symmetric and Anti-symmetric Forces

Define

92 $$\begin{aligned} F^S(\rho ) := \frac{1}{2}[ F(\rho ) + F^*(\rho ) ], \qquad F^A(\rho ) := \frac{1}{2}[ F(\rho ) - F^*(\rho ) ]. \end{aligned}$$

As the following proposition shows, $F^S$ is connected to the gradient of the free energy (or quasipotential) $I_0$, and the forces $F^A$ and $F^S$ satisfy a generalised orthogonality (in the sense of Proposition 3). The proof follows Section II.C of [7], but uses only the assumptions of Sect. 6.1, showing that the result applies also to Markov chains.

#### Proposition 11

The forces $F^S$ and $F^A$ satisfy

93 $$\begin{aligned} F^S(\rho ) = -\nabla \frac{\delta I_0}{\delta \rho }, \end{aligned}$$

and

94 $$\begin{aligned} \varPsi ^\star \bigl (\rho , F^S(\rho ) + F^A\bigr ) = \varPsi ^\star \bigl (\rho , F^S(\rho ) - F^A\bigr ). \end{aligned}$$

#### Proof

Combining (90) and (91), we obtain (for any path $(\rho _t,j_t)_{t\in [0,T]}$ that obeys the continuity equation $\dot{\rho } = -{\mathrm{div}\,}j$)

95 $$\begin{aligned} I_0(\rho _0) + \frac{1}{2} \int _0^T\varPhi (\rho _t,j_t,F(\rho _t))\,{\mathrm {d}}t =I_0(\rho _T) + \frac{1}{2}\int _0^T \varPhi (\rho _{T-t},-j_{T-t},F^*(\rho _{T-t}))\,{\mathrm {d}}t. \end{aligned}$$

Differentiating with respect to *T* and using (3) together with $\varPsi (\rho ,j)=\varPsi (\rho ,-j)$ and (92), one has

$$\begin{aligned} \dot{I}_0(\rho ) + j\cdot F^S(\rho ) + \frac{1}{2} \big [ \varPsi ^\star (\rho ,F^*(\rho )) - \varPsi ^\star (\rho ,F(\rho )) \big ] = 0. \end{aligned}$$

Using the continuity equation and an integration by parts, one finds $\dot{I}_0(\rho ) =j\cdot \nabla \frac{\delta I_0}{\delta \rho }$, so that

$$\begin{aligned} j\cdot \left[ F^S(\rho ) + \nabla \frac{\delta I_0}{\delta \rho } \right] + \frac{1}{2} \big [ \varPsi ^\star (\rho ,F^*(\rho )) - \varPsi ^\star (\rho ,F(\rho )) \big ] = 0. \end{aligned}$$

This equation must hold for all $(\rho ,j)$, which means that the two terms in square parentheses both vanish separately. Combining the last equation with (92), we obtain (93) and (94). $\square$

Proposition 11 also yields a variational characterisation of $I_0$. The following corollary is analogous to Eq. (4.8) of [7], as is its proof.

#### Corollary 12

The free energy $I_0$ satisfies

96 $$\begin{aligned} I_0(\hat{\rho }) = \inf { \frac{1}{2}\int _{-\infty }^0 \varPhi (\rho _t,j_t, F(\rho _t)) \,{\mathrm {d}}t }, \end{aligned}$$

where the infimum is taken over all paths $(\rho _t, j_t)_{t\in (-\infty ,0]}$ that satisfy $\dot{\rho }_t+{\mathrm{div}\,}j_t=0$, as well as $\lim _{t\rightarrow -\infty }\rho _t = \pi$ and $\rho _0 = \hat{\rho }$. Moreover, the optimal path is given by the time reversal of the solution of the adjoint dynamics $(\rho _t, -J^*(\rho _t))_{t\in (-\infty ,0]}$.

#### Proof

We obtain from (95) (together with (2) and (90)) that

$$\begin{aligned} \frac{1}{2}\int _{-\infty }^0 \varPhi (\rho _t,j_t, F(\rho _t)) \,{\mathrm {d}}t = I_0(\hat{\rho }) + \frac{1}{2}\int _{-\infty }^0 \varPhi (\rho _t,j_t, F^*(\rho _t)) \,{\mathrm {d}}t. \end{aligned}$$

Taking the infimum on both sides yields (96); indeed the infimum of $\frac{1}{2}\int _{-\infty }^0 \varPhi (\rho _t,j_t, F(\rho _t)) \,{\mathrm {d}}t$ is 0, and this infimum is attained uniquely for the optimal path for (96). To see this, we note that $\varPhi (\rho _t,-j_t, F^*(\rho _t))$ is uniquely minimised for $j_t = -J^*(\rho _t)$, and $(\rho _t, -J^*(\rho _t))_{t\in (-\infty ,0]}$ satisfies the conditions above, so the optimal path is indeed the time-reversal of the solution of the adjoint dynamics. $\square$

### Hamilton–Jacobi Like Equation for the Extended Hamiltonian

Another important relationship within MFT is the Hamilton–Jacobi equation [7, Eq. (4.13)]. This provides a characterisation of the quasipotential, as its maximal non-negative solution. The following formulation of that result uses only the assumptions of Sect. 6.1 and therefore applies also to Markov chains. The functional

97 $$\begin{aligned} \mathbb {L}(\rho ,j):=\frac{1}{2} \varPhi (\rho ,j,F(\rho )) \end{aligned}$$

can be interpreted as an extended Lagrangian. (Note that $\mathbb {L}(\rho ,j)$ should not be interpreted as a Lagrangian in the classical sense, as it depends on density and current $(\rho ,j)$, rather than the pair consisting of density and associated velocity $(\rho ,\dot{\rho })$). We follow Sect. IV.G of [7]: given a sample path $(\rho _t,j_t)_{t\in [0,T]}$), define a vector field $A_t=A_0 - \int _0^t j_s \mathrm {d}s$. The initial condition $A_0$ is chosen so that there is a bijection between the paths $(\rho _t,j_t)_{t\in [0,T]}$ and $(A_t)_{t\in [0,T]}$. For example, in finite Markov chains, define $\bar{\rho }$ as a constant density, normalised to unity, and let $A_0=\nabla h$, where *h* solves ${\mathrm{div}\,}(\nabla h) = (\rho _0-\bar{\rho })$, see [13] for the relevant properties of these vector fields. With this choice, and using $\dot{\rho } = -{\mathrm{div}\,}j$, one has $\rho _t=\bar{\rho }+ {\mathrm{div}\,}A_t$ for all *t*, and one may also write (formally) $A_t = {\mathrm{div}\,}^{-1}(\rho _t-\bar{\rho })$. Comparing with [7, Sect. IV.G], we write $\rho =\bar{\rho }+{\mathrm{div}\,}A$ instead of $\rho ={\mathrm{div}\,}A$ since for Markov chains one has (for any discrete vector field *A*) that $\sum _x {\mathrm{div}\,}A(x)=0$, so it is not possible to solve ${\mathrm{div}\,}A = \rho$ if $\rho$ is normalised to unity (recall that discrete vector fields have by definition $A_{xy}=-A_{yx}$ [13]).

The fluctuations of *A* are therefore determined by the fluctuations of $(\rho ,j)$, so the LDP (1) implies a similar LDP for *A*, whose rate function is $I^\mathrm{ex}_{[0,T]}((A_t)_{t\in [0,T]}) = I^\mathrm{ex}_0(A_0) + \int _0^T \mathbb {L}^\mathrm{ex}(A_t,\dot{A}_t)\mathrm {d}t$, where $\mathbb {L}^\mathrm{ex}$ is a Lagrangian that depends on *A* and its time derivative (which we again refer to as extended Lagrangian, cf. [7]). The function $\mathbb {L}$ in (97) is then related to $\mathbb {L}^\mathrm{ex}$ via the bijection between $(\rho ,j)$ and *A*. Considering again the case of Markov chains, the time evolution of the system depends only on ${\mathrm{div}\,}A$ (which is $\rho -\bar{\rho }$) and not on *A* itself, one sees that $\mathbb {L}^\mathrm{ex}(A,\dot{A})$ depends only on ${\mathrm{div}\,}A$ and $\dot{A}$ (which is *j*). Hence we write, formally, $\mathbb {L}(\rho ,j) = \mathbb {L}^\mathrm{ex}({\mathrm{div}\,}^{-1}(\rho -\bar{\rho }),-j)$, and we recover (97).

Hence $\mathbb {L}$ is nothing but the extended Lagrangian $\mathbb {L}^\mathrm{ex}$, written in different variables: for this reason we refer to $\mathbb {L}$ as an (extended) Lagrangian.

To arrive at the corresponding (extended) Hamiltonian, one should write $\mathbb {H}^\mathrm{ex}(A,\xi ) = \sup _{\dot{A}} [ \xi \cdot \dot{A} - \mathbb {L}^\mathrm{ex}(A_t,\dot{A}_t) ]$, or equivalently

98 $$\begin{aligned} \mathbb {H}(\rho ,\xi ) =\sup _j \bigl ( j \cdot \xi - \mathbb {L}(\rho ,j)\bigr ), \end{aligned}$$

where $\xi$ is a conjugate field for the current *j*. We identify $\mathbb {H}$ as the scaled cumulant generating function associated with the rate function $I_{2.5}(\rho ,j)=\mathbb {L}(\rho ,j)$ [52, Sect. 3.1]. Analysis of rare fluctuations in terms of the field $\xi$ is often more convenient than direct analysis of the rate function [32, 33] and is the basis of the “*s*-ensemble” method that has recently been exploited in a number of physical applications (for example [21, 24]). Using (3) and (4), we obtain

99 $$\begin{aligned} \mathbb {H}(\rho ,\xi ) =\frac{1}{2}\varPsi ^\star (\rho ,F(\rho )+2\xi ) - \frac{1}{2}\varPsi ^\star (\rho ,F(\rho )). \end{aligned}$$

(This generalises the definition (44), which was restricted to Markov chains.)

To relate this extended Hamiltonian to the free energy (quasipotential), one can define an *extended Hamilton–Jacobi equation*, which is for a functional $\mathcal {S}$ given by

100 $$\begin{aligned} \mathbb {H}\left( \rho ,\nabla \frac{\delta \mathcal {S}}{\delta \rho }\right) =0. \end{aligned}$$

The relation of this equation to the free energy is given by the following proposition, which mirrors equation (4.18) of [7], but now in our generalised setting, so that it applies also to Markov chains.

#### Proposition 13

The free energy $I_0$ is the maximal non-negative solution to (100) which vanishes at the steady state $\pi$. In other words, any functional $\mathcal {S}$ that solves (100) and has $\mathcal {S}(\pi )=0$ also satisfies $\mathcal {S}\le I_0$.

#### Proof

From (92), (93), (94) and $\varPsi ^\star (\rho ,F)=\varPsi ^\star (\rho ,-F)$, one has

101 $$\begin{aligned} \varPsi ^\star \left( \rho ,F(\rho )+2\nabla \tfrac{\delta I_0}{\delta \rho }\right) =\varPsi ^\star (\rho ,-F_S(\rho )+F_A(\rho ))=\varPsi ^\star (\rho ,F(\rho )). \end{aligned}$$

Thus (99) yields $\mathbb {H}\bigl (\rho ,\nabla \tfrac{\delta I_0}{\delta \rho }\bigr )=0$, so $I_0$ does indeed solve (100). In addition, (101) is valid also with $I_0$ replaced by any $\mathcal {S}$ that solves (100); combining this result with (3) yields

102 $$\begin{aligned} \varPhi (\rho ,j,F(\rho )) = \varPhi \left( \rho ,j,F(\rho ) +2\nabla \frac{\delta \mathcal {S}}{\delta \rho }\right) + 2 j\cdot \nabla \frac{\delta \mathcal {S}}{\delta \rho }\ge 2 j\cdot \nabla \frac{\delta \mathcal {S}}{\delta \rho }, \end{aligned}$$

where the second step uses $\varPhi \ge 0$. Moreover, for any path $(\rho _t,j_t)_{t\in (-\infty ,0]}$ with $\dot{\rho }_t+{\mathrm{div}\,}j_t=0$ and $\lim _{t\rightarrow -\infty }\rho _t = \pi$, we have from (102) that

$$\begin{aligned}&I_{(-\infty ,0]}\bigl ((\rho ,j)_{t\in (-\infty ,0]}\bigr ) = \int _{-\infty }^0 \varPhi (\rho _t,j_t,F(\rho _t)) \,{\mathrm {d}}t \\&\quad \ge \int _{-\infty }^0 j(x)\cdot \nabla \frac{\delta \mathcal {S}}{\delta \rho }(x) \,{\mathrm {d}}t = \mathcal {S}(\rho _0),\qquad \end{aligned}$$

where the final equality uses an integration by parts, together with the continuity equation. Finally, taking the infimum over all paths and using Corollary 12, one obtains $\mathcal {S}(\rho ) \le I_0(\rho )$, as claimed. $\square$

### Generalisation of Lemma 2

Before ending, we note that (94) is analogous to Proposition 3 in the general setting of this section, but we have not yet proved any analogue of Lemma 2. Hence we have not obtained a generalisation of Corollary 4, nor any of its further consequences. To achieve this, one requires a further assumption within the general framework considered here, which amounts to a splitting of the Hamiltonian. This assumption holds for MFT and for Markov chains, and is a sufficient condition for a generalised Lemma 2.

To state the assumption, we consider a reversible process in which the forces are $F^S(\rho )$. (For Markov chains we should consider the process with rates $r^S_{xy} = \frac{1}{2}( r_{xy} + r_{xy}^*)$; for MFT it is the process with $J(\rho )=J^S(\rho )$ and the same mobility $\chi$ as the original process.) We assume that such a process exists and that its Hamiltonian can be written as $\mathbb {H}_S(\rho ,\xi ) = \frac{1}{2} [ \varPsi ^\star _S(\rho ,F^S(\rho ) + 2\xi ) - \varPsi _S^\star (\rho ,F^S(\rho ))]$ for some function $\varPsi ^\star _S$ (compare (99) and see Sect. 3.4 for the case of Markov chains). Also let the Hamiltonian for the adjoint process be $\mathbb {H}^*(\rho ,\xi )$, which is constructed by replacing *F* by $F^*$ in (99). Then, one assumes further that

103 $$\begin{aligned} \mathbb {H}_S(\rho ,\xi ) = \tfrac{1}{2} [ \mathbb {H}(\rho ,\xi ) + \mathbb {H}^*(\rho ,\xi ) ], \end{aligned}$$

which may be verified to hold for Markov chains and for MFT. Writing $\xi =-F^S/2$ and using (99) with (94) and $\varPsi ^\star (\rho ,f) = \varPsi ^\star (\rho ,-f)$, one then obtains

104 $$\begin{aligned} \varPsi ^\star _S(\rho ,F^S(\rho )) = \varPsi ^\star (F(\rho )) - \varPsi ^\star (F^A(\rho )), \end{aligned}$$

which is the promised generalisation of Lemma 2.

## Conclusion

In this article, we have presented several results for dynamical fluctuations in Markov chains. The central object in our discussion has been the function $\varPhi$, which plays a number of different roles—it is the rate function for large deviations at level 2.5 (Eq. 64), and it also appears in the rate function for pathwise large deviation functions (Eq. 2). These results—derived originally by Maes et al. [38, 39]—originate from the relationship between $\varPhi$ and the relative entropy between path measures (Appendix A). The canonical (Legendre transform) structure of $\varPhi$ (Eq. 4) and its relation to time reversal (Eq. 25) have also been discussed before [38].

The function $\varPhi$ depends on probability currents *j* and their conjugate forces *f*. Our Proposition 3 and Corollary 4 show how the rate functions in which $\varPhi$ appears have another level of structure, based on the decomposition of the forces *F* in two pieces $F=F^S+F^A$, according to its behaviour under time-reversal. A similar decomposition is applied in MFT [7]: the discussion of Sects. 5 and 6 show how several results of that theory—which applies on macroscopic (hydrodynamic) scales—already have analogues for Markov chains, which provide microscopic descriptions of interacting particle systems. These results—which concern symmetries, gradient structures and (generalised) orthogonality relationships—show how properties of the rate functions are directly connected to physical ideas of free energy, dissipation, and time-reversal.

Looking forward, we hope that these structures can be exploited both in mathematics and physics. From a mathematical viewpoint, the canonical structure and generalised orthogonality relationships may provide new routes for scale-bridging calculations, just as the geometrical structure identified by Maas [35] has been used to develop new proofs of hydrodynamic limits [17]. In physics, a common technique is to propose macroscopic descriptions of physical systems based on symmetries and general principles—examples in non-equilibrium (active) systems include [51, 54]. However, this level of description leaves some ambiguity as to the best definitions of some physical quantities, such as the local entropy production [44]. We hope that the structures identified here can be useful in relating such macroscopic theories to underlying microscopic behaviour.

## Appendix A: Relative Entropy on Path Space

Consider a Markov process with rates *r*(*x*, *y*) and initial distribution $Q_0$. We fix a time interval [0, *T*] for some $T>0$ and denote the distribution of the Markov process on this time interval with *Q*. For each path $(x_u)_{u\in [0,T]}$ with jumps at times $t_1,\dots , t_n$ the density of *Q* can be found by solving the associated master equation (11); it is given by

$$\begin{aligned} Q\bigl ( (x_u)_{u\in [0,T]}\bigr ) = Q_0(x_0)\exp \biggl \{\int _0^T \biggl (\sum _{i=1}^n\log r_t(x_{t-},x_t) \delta (t-t_i) - \sum _y r_t(x_t,y) \biggr )\,{\mathrm {d}}t\biggr \}, \end{aligned}$$

where $x_{t-}:=\lim _{\epsilon \rightarrow 0}x_{t-\epsilon }$ is the state of the process just before time *t*.

Now consider a second Markov process with time-dependent rates $\hat{r}_t(x,y)$ and initial distribution $P_0$. The distribution of this process is denoted by *P*. The logarithmic density of *P* with respect to *Q* is given by

$$\begin{aligned}&\log \frac{dP}{dQ}\bigl ((x_u)_{u\in [0,T]}\bigr ) =\log \frac{dP_0}{dQ_0}(x_0) \\&\quad + \int _{0}^{T} \biggl (\sum _{i=1}^n\log \Bigl (\frac{\hat{r}_t(x_{t^-},x_{t})}{r(x_{t^-},x_{t})}\Bigr )\;\! \delta (t-t_i) - \sum _y \bigl [\hat{r}_t(x_t,y)-r(x_t,y)\bigr ]\biggr )\,{\mathrm {d}}t. \end{aligned}$$

We further denote the distribution of *P* at time *t* with $\rho _t$, such that $\rho _t = P\circ X_t^{-1}$ where $X_t$ denotes the evaluation of the path at time *t* (such that in particular $P_0=\rho _0$). The *relative entropy* on path space

$$\begin{aligned} \mathcal {H}(P|Q) := \mathbb {E}_P\biggl [\log \Bigl (\frac{dP}{dQ}\Bigr )\biggr ] \end{aligned}$$

is then equal to

$$\begin{aligned} \mathbb {E}_{P_0}\biggl [\log \Bigl (\frac{dP_0}{dQ_0}\Bigr )\biggr ] + \int _0^T \sum _{x,y}\rho _t(x)\Bigl (\hat{r}_t(x,y) \log \Bigl (\frac{\hat{r}_t(x,y)}{r(x,y)}\Bigr )-\hat{r}_t(x,y)+r(x,y)\Bigr )\,{\mathrm {d}}t. \end{aligned}$$

Let $(\rho _t,j_t)_{t\in [0,T]}$ be given, with $\rho _t>0$ for all times $t\in [0,T]$. We then can rewrite the relative entropy $\mathcal {H}(P|Q)$ in terms of the flow $C_t(x,y):=\rho _t(x)\hat{r}_t(x,y)$ as

105 $$\begin{aligned} \mathcal {H}(\rho _0|Q_0) + \int _0^T\sum _{x,y}\Bigl (C_t(x,y)\log \Bigl (\frac{C_t(x,y)}{\rho _t(x)r(x,y)}\Bigr ) - C_t(x,y) + \rho _t(x)r(x,y) \Bigr )\,{\mathrm {d}}t. \end{aligned}$$

Note that the relative entropy $\mathcal {H}(P|Q)$ can (just as the Markov chain) be completely characterised by the probability distribution $(\rho _t)_{t\in [0,T]}$ and the flow $(C_t)_{t\in [0,T]}$.

We are interested in a special flow $(C_t)_{t\in [0,T]}$ which recovers a given current $(j_t)_{t\in [0,T]}$ as $(j_t)_{xy}=C_t(x,y)-C_t(y,x)$. The force associated to $j_t$ is by (13) given by $f^{j_t}(\rho _t):=2 {\text {arcsinh}}(j_t/a(\rho _t))$ and the flow of interest is defined as $C_t(x,y) = \frac{1}{2} a_{xy}(\rho _t) \exp (\frac{1}{2} f_{xy}^{j_t}(\rho _t))$. It can be interpreted as the optimal flow that creates the current $(j_t)_{t\in [0,T]}$.

We define the rates $\tilde{r}_t(x,y):=C_t(x,y)/\rho _t(x)$ and denote the law of the associated (time heterogeneous) Markov process on [0, *T*] with $\tilde{P}$. The relative entropy of this new process $\tilde{P}$ with respect to the reference process *Q* is

106 $$\begin{aligned} \mathcal {H}(\tilde{P} | Q) = \mathcal {H}(\rho _0|Q_0) + \frac{1}{2} \int _0^T \varPhi (\rho _t,j_t, F(\rho _t)) \,{\mathrm {d}}t \end{aligned}$$

with $\varPhi$ given by (3); to see this, we argue as follows. Symmetrising (105) and considering each summand separately gives

$$\begin{aligned}&\frac{1}{2}\Bigl ( C_t(x,y)\log \frac{C_t(x,y)}{C_t^Q(x,y)} + C_t(y,x)\log \frac{C_t(y,x)}{C_t^Q(y,x)} \Bigr ) \\&\quad + \frac{1}{2} \Bigl ( C_t^Q(x,y) - C_t(x,y) + C_t^Q(y,x) - C_t(y,x) \Bigr ),\qquad \end{aligned}$$

where the first summand coincides with

$$\begin{aligned} \frac{1}{2} \biggl ( \frac{1}{2} a_{xy}(\rho _t)\sinh \bigl (\tfrac{1}{2}f^{j_t}_{xy}(\rho _t)\bigr ) f^{j_t}_{xy}(\rho _t) - \frac{1}{2} a_{xy}(\rho _t)\sinh \bigl (\tfrac{1}{2} f^{j_t}_{xy}(\rho _t)\bigr ) F_{xy}(\rho _t)\biggr ) \end{aligned}$$

and the second is given by

$$\begin{aligned} \frac{1}{2} \Bigl (a_{xy}(\rho _t)\cosh \bigl (\tfrac{1}{2}F_{xy}(\rho _t)\bigr ) -a_{xy}(\rho _t)\cosh \bigl (\tfrac{1}{2}f^{j_t}_{xy}(\rho _t)\bigr )\Bigr ). \end{aligned}$$

Combining this with (15) and (16) yields (106).

*Pathwise Large Deviation Principle*: Let $x^1,x^2,\dots$ be a sequence of iid copies of the Markov chains with law *Q*. By Sanov’s Theorem (see, e.g., Theorem 6.2.10 in [14]), the empirical average $\frac{1}{\mathcal {N}}\sum _{i=1}^\mathcal {N} \delta _{x^i}$ of the Markov chains satisfies a LDP with the rate functional $\mathcal {H}(\cdot | Q)$. We can interpret $\mathcal {H}(\cdot | Q)$ as the rate functional for the joint LDP of $(\rho _t,C_t)_{t\in [0,T]}$ by defining this rate functional $\mathcal {I}_{[0,T]}((\rho _t,C_t)_{t\in [0,T]})$ as the right-hand side of (105).

We contract the above rate functional to obtain the rate functional for the joint empirical measure and current $(\rho _t,j_t)_{t\in [0,T]}$. It is given by

107 $$\begin{aligned} I_{[0,T]}((\rho _t,j_t)_{t\in [0,T]}):=\inf _{(C_t)_{t\in [0,T]}} \mathcal {I}_{[0,T]}((\rho _t,C_t)_{t\in [0,T]}), \end{aligned}$$

where the infimum is taken over the set of all flows which yield the current $(j_t)_{t\in [0,T]}$, i.e. over the set $\{(C_t)_{t\in [0,T]} |$ for all $t\in [0,T]: C_t(x,y)\ge 0$ and $C_t(x,y) - C_t(y,x) = (j_t)_{xy}\}$. It was shown in [38] and [9] that the minimising flow is the current $C_t(x,y) = \frac{1}{2} a_{xy}(\rho _t) \exp (\frac{1}{2} f_{xy}^{j_t}(\rho _t))$ introduced above, such that $I_{[0,T]}((\rho _t,j_t)_{t\in [0,T]})$ coincides with (106).
